# Predicting In‐Hospital Mortality in Patients With Acute Myocardial Infarction: A Comparison of Machine Learning Approaches

**DOI:** 10.1002/clc.70124

**Published:** 2025-03-27

**Authors:** Hamidreza Soleimani, Soroush Najdaghi, Delaram Narimani Davani, Parham Dastjerdi, Parham Samimisedeh, Hedieh Shayesteh, Babak Sattartabar, Farzad Masoudkabir, Haleh Ashraf, Mehdi Mehrani, Yaser Jenab, Kaveh Hosseini

**Affiliations:** ^1^ Tehran Heart Center, Cardiovascular Disease Research Institute Tehran University of Medical Sciences Tehran Iran; ^2^ Heart Failure Research Center, Cardiovascular Research Institute Isfahan University of Medical Sciences Isfahan Iran; ^3^ Clinical Cardiovascular Research Center Alborz University of Medical Sciences Karaj Alborz Iran

**Keywords:** acute myocardial infarction, in‐hospital mortality, machine learning, random forest

## Abstract

**Background:**

Acute myocardial infarction (AMI) remains a leading global cause of mortality. This study explores predictors of in‐hospital mortality among AMI patients using advanced machine learning (ML) techniques.

**Methods:**

Data from 7422 AMI patients treated with percutaneous coronary intervention (PCI) at Tehran Heart Center (2015–2021) were analyzed. Fifty‐eight clinical, demographic, and laboratory variables were evaluated. Seven ML algorithms, including Random Forest (RF), logistic regression with LASSO, and XGBoost, were implemented. The data set was divided into training (70%) and testing (30%) subsets, with fivefold cross‐validation. The class imbalance was addressed using the synthetic minority oversampling technique (SMOTE). Model predictions were interpreted using SHapley Additive exPlanations (SHAP).

**Results:**

In‐hospital mortality occurred in 129 patients (1.74%). RF achieved the highest predictive performance, with an area under the curve (AUC) of 0.924 (95% CI 0.893–0.954), followed by XGBoost (AUC 0.905) and logistic regression with LASSO (AUC 0.893). Sensitivity analysis in STEMI patients confirmed RF's robust performance (AUC 0.900). SHAP analysis identified key predictors, including lower left ventricular ejection fraction (LVEF; 33.24% vs. 43.46% in survivors, *p* < 0.001), higher fasting blood glucose (190.38 vs. 132.29 mg/dL, *p* < 0.001), elevated serum creatinine, advanced age (70.92 vs. 61.88 years, *p* < 0.001), and lower LDL‐C levels. Conversely, BMI showed no significant association (*p* = 0.456).

**Conclusion:**

ML algorithms, particularly RF, effectively predict in‐hospital mortality in AMI patients, highlighting critical predictors such as LVEF and biochemical markers. These insights offer valuable tools for enhancing clinical decision‐making and improving patient outcomes.

## Graphical abstract

In this study, we developed and validated machine learning models to predict in‐hospital mortality in patients with myocardial infarction undergoing PCI, using data from Tehran Heart Center (2011–2020). Our data set included 7422 patients, with 129 cases of in‐hospital mortality, and was built from 41 routinely collected clinical variables. We trained and evaluated seven machine learning models, identifying Random Forest as the best‐performing model, achieving an AUC of 0.92, sensitivity of 0.97, and specificity of 0.75. Our analysis revealed that LVEF and creatinine levels were the most significant predictors of in‐hospital mortality.

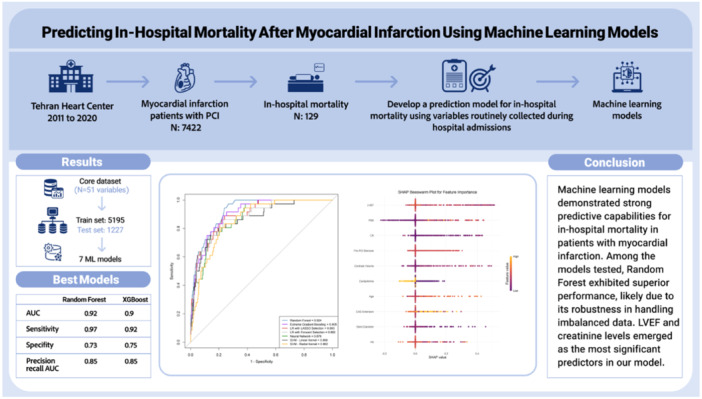

**Abbreviations**
3VD: three‐vessel disease
ACC: American College of Cardiology
ACTION‐GWTG: Acute Coronary Treatment and Intervention Outcomes Network—Get With The Guidelines
AMI: acute myocardial infarction
AUC: area under the curve
AUC‐PR: area under the curve precision‐recall
BMI: body mass index
CAD: coronary artery disease
CKD: chronic kidney disease
eGFR: estimated glomerular filtration rate
FBS: fasting blood sugar
GRACE: Global Registry of Acute Coronary Events
HDL‐C: high‐density lipoprotein cholesterol
HFrEF: heart failure with reduced ejection fraction
IRB: Institutional Review Board
KNN: k‐nearest neighbor
LAD: left anterior descending
LASSO: Least Absolute Shrinkage and Selection Operator
LDL‐C: low‐density lipoprotein cholesterol
LVEF: left ventricular ejection fraction
MI: myocardial infarction
ML: machine learning
NN: neural network
NSTEMI: non‐ST‐segment elevation myocardial infarction
PCI: percutaneous coronary intervention
PRC: precision‐recall curve
RF: Random Forest
ROC: Receiver operating characteristic
SHAP: SHapley Additive exPlanations
SMOTE: synthetic minority oversampling technique
STEMI: ST‐segment elevation myocardial infarction
SVM: support vector machine
THC: Tehran Heart Center
TIMI: Thrombolysis in Myocardial Infarction
XGBoost: eXtreme Gradient Boosting

## 1 Introduction

Acute myocardial infarction (AMI) is still the leading cause of mortality worldwide [1], with significant mortality occurring during initial hospitalization or within the first month post‐MI [2, 3]. Despite advances in reperfusion treatments, which have notably reduced in‐hospital mortality over the past decade [4], substantial risk remains, particularly among high‐risk patients, including those with cardiogenic shock or advanced age [5]. Early and optimal risk stratification to identify high‐risk individuals has been shown to improve clinical outcomes and reduce morbidity in patients with MI [6]. Several risk stratification tools have been developed to predict in‐hospital mortality, aiding prognosis assessment and enabling individualized treatment strategy, care level, and hospital duration for MI patients [7]. The Global Registry of Acute Coronary Events (GRACE) and Thrombolysis in Myocardial Infarction (TIMI) risk scores are widely applied to assess both in‐hospital and short‐term mortality following MI [7, 8]. However, their predictive accuracy has become limited in current clinical practice due to several drawbacks: since their introduction, advancements in interventional reperfusion strategies have significantly improved patient outcomes, rendering these models less effective [9]. Moreover, these scores do not account for many variables now recognized as independent predictors of mortality in acute MI. For instance, certain angiographic features—such as multiple complex coronary lesions [10], left main trunk or proximal left anterior descending (LAD) involvement as the culprit's vessel, and lower TIMI myocardial perfusion grade [11]—are strongly associated with poorer outcomes, yet are not included in these scores.

Machine learning (ML)'s ability to analyze large data sets, integrate numerous variables, and explore complex interactions, including nonlinear relationships, makes it a highly efficient tool for improving the accuracy of risk stratification and outcome prediction [12]. Despite efforts to improve the predictive accuracy of traditional risk stratification methods through the addition or modification of variables, these scores continue to underperform when compared to more recent ML‐based models [13, 14]. A pooled analysis of randomized clinical trials, involving a large cohort of patients with acute coronary syndrome, demonstrated the superior performance of ML models over traditional risk stratification methods [15].

Several studies have focused on developing ML‐based predictive models for in‐hospital mortality in patients with acute MI [16, 17]. However, there are key issues that need to be addressed to ensure these models are practical, generalized, and clinically relevant. Specifically, ML models should be built using variables that are routinely available during the acute course of MI. Introducing and using variables that are not typically collected in clinical practice can limit the model's applicability and integration into standard care. Moreover, different ML models are designed for distinct purposes. For example, when dealing with large, imbalanced data sets—such as those used for predicting in‐hospital mortality post‐MI—specific models like Random Forests (RF) are particularly suitable due to their ability to handle complex data structures and nonlinear relationships [18]. Furthermore, in clinical prediction tasks, eXtreme Gradient Boosting (XGBoost)—a widely used gradient‐boosting algorithm—is particularly effective [19] for its ability to efficiently process large clinical data sets, handle missing data, and prevent overfitting through built‐in regularization techniques [20]. In this study, we prioritized the use of simple, feasible, and commonly obtained variables to develop and train our ML models. To the best of our knowledge, this is the first study utilizing ML models for predicting in‐hospital mortality in Iranian patients with AMI, leveraging available in‐hospital data and relevant ML algorithms.

## 2 Methods

### 2.1 Study Population

For this study data from Tehran Heart Center's (THC) electronic registry were accessed. All consecutive patients admitted with AMI, including both ST‐segment elevation myocardial infarction (STEMI) and non‐ST segment AMI (NSTEMI), who had been hospitalized and undergone percutaneous coronary intervention (PCI) between 2015 and 2021, were eligible for inclusion.

### 2.2 Candidate Variables

THC's electronic registry records demographic, laboratory, procedural, and clinical outcome data from patients. For this study, all the above‐mentioned variables were examined for the number of missing data. All variables with more than 10% missing data were removed, also variables that only had one value, and variables with zero or near zero variance were omitted. At the end of this process, 58 candidate variables remained which were selected for model development (16 numeric and 42 categorical). These variables included baseline demographics, clinical characteristics, and features related to the medical history of the patient (*n* = 26), laboratory data (*n* = 7), left ventricular ejection fraction (LVEF) (*n* = 1), invasive coronary angiography data (*n* = 10), and features relating to intra‐ or postprocedural complications (*n* = 14). For a list of fully selected variables and their definitions, please see Table S1.

### 2.3 Statistical Analysis

Baseline characteristics in this study were reported as mean ± standard deviation (SD) for continuous variables and percentage and number of events for categorical variables. To assess the normal distribution of data Kolmogorov–Smirnov test was utilized. To compare feature variables between two groups of patients (those with and without in‐hospital mortality, respectively), Pearson's *χ* ^2^ and Fisher's exact test were used for categorical variables and either Student's *t*‐test (normal distribution) or Mann–Whitney *U* test (skewed distribution) was used for comparison of continuous variables. All statistical analysis, model developments, and data summary and visualization for this study were conducted with R software [21] (R for Windows, Version 4.1.3, Vienna, Austria) and R Studio Version 1.1.463 (Posit PBC, Boston, MA, USA). For a list of packages used in this study please see Supporting Information (Statistical Package) [22, 23, 24, 25, 26, 27, 28, 29, 30, 31, 32, 33].

### 2.4 Data Processing

We applied range normalization to continuous variables to adjust their values to a scale between 0 and 1, thereby reducing the risk of model bias due to the varying magnitudes of the numerical data. For categorical variables, we utilized one‐hot encoding, a method that converts categorical data into pairs of binary values (0 and 1) to make the variables suitable for use in classification algorithms [34]. The data set was then examined for missing data. To handle missing data, we used Multiple Imputation by Chained Equations, which enabled the retention of variables with missing values. The THC data set assigns a unique code to each patient, ensuring that all associated data are consistently linked to this identifier. As a result, no duplicate entries were present in the data set, and the data were verified to be free of duplicates.

### 2.5 Model Development

The study population was randomly divided into training and testing subsets with a 70%/30% proportion. To ensure a fair and representative distribution of the outcome of in‐hospital mortality, the split was stratified based on the target variable, maintaining a balanced proportion of mortality events in both data sets. This approach minimizes bias and ensures that both subsets accurately reflect the overall distribution of in‐hospital mortality.

To avoid overfitting, for each model, we conducted fivefold cross‐validation by randomly dividing the training data set into five segments for a total of five iterations. In each iteration, we used four segments for training and one segment for internal validation. Then this final model was tested on the test data set (30% of the entire data), which remained unseen throughout the entire model development process. Furthermore, to assess the reliability of our predicted probabilities, we generated calibration plots for both the main analysis and the mixed model. These plots evaluate the agreement between predicted and observed event probabilities, providing an essential measure of model calibration. A well‐calibrated model ensures that predicted risks accurately reflect actual outcomes, reducing concerns about overfitting.

In this study, seven widely accepted and commonly used ML algorithms were utilized. These algorithms included: logistic regression with forward variable selection, logistic regression with Least Absolute Shrinkage and Selection Operator (LASSO) regularization, support vector machine (SVM) with linear and radial kernels, a single layer neural network (NN) model, RF, and XGBoost.

Logistic regression with forward stepwise selection was used to identify statistically significant variables (*p* < 0.05) through a likelihood ratio test, assessing incremental improvements in the C‐statistic. In high‐dimensional cases, forward selection shows robustness against noise, unlike backward selection [35, 36, 37]. LASSO‐regularized logistic regression helps create a concise model by enforcing stricter criteria for variable inclusion [38]. RF techniques build multiple decision trees to categorize patients by significant variables, using a voting mechanism to produce predictions. Gradient boosting selects decision tree variables that best predict outcomes. Boosting has become more prevalent in ML as it entails the stepwise development of models, where each new iteration aims to address the mistakes made by the previous model [39]. SVM establishes a boundary between two classes using linear and radial‐based kernels to identify variable relationships. A single‐layer NN (perceptron) was employed for binary classification, learning via gradient descent to adjust weights and minimize prediction error. The model uses an output layer driven by a threshold activation function to classify data into two distinct categories effectively. To tune the parameters of each model grid search with fivefold cross‐validation was performed.

To address the significant imbalance in the prevalence of the target variable, a random synthetic minority oversampling technique (SMOTE) was used, which identifies the KNN of the minority group and creates new data based on them. When contrasted with undersampling, another method for managing imbalanced data, SMOTE oversampling is considered more advantageous because it retains important data [40, 41, 42]. A sensitivity analysis was performed where all models were trained and tested without the use of SMOTE oversampling, results of this comparison are presented in the Supporting Information.

In addition to the ML models, a mixed model was developed by combining the GRACE score with the top 5 predictors identified in this study, ranked by feature importance. The performance of this mixed model was evaluated in terms of AUC, AUC‐PR, and Brier score to compare it with the ML models, demonstrating the incremental value of ML‐based approaches and effectively benchmarking their performance.

### 2.6 Evaluating Model Performance

Receiver Operating Characteristic (ROC) Curve were utilized to assess each model's ability to distinguish between two classes of patients. For each model, area under the curve (AUC), sensitivity, and specificity were calculated and reported. For the top 4 performing models we constructed precision‐recall curves (PRC) and reported the area under this curve (AUC‐PR). In short, PRC plots can offer the viewer a reliable forecast of future classification performance since they assess the proportion of true positives within positive predictions and it has been proposed that this metric might offer more accurate information regarding the performance of classification algorithms especially when dealing with imbalanced data sets [43].

### 2.7 Top Predictors

To make the top‐performing model explainable, the importance of each variable was assessed. This assessment differs in each model, for example in RF models, a mean decrease in accuracy is used to rank the importance of variables, and gradient boosting algorithms identify the most important variables based on the number of trees a variable appears in and the information gain that it provides [37]. SHapley Additive exPlanations (SHAP) is a game theoretic approach that quantifies and explains the contribution of each variable to the final model output. It links the best distribution of credit with localized explanations by utilizing traditional Shapley values from game theory and their associated adaptations [44]. SHAP values and beeswarm plots of feature importance were also generated for the top‐performing model.

### 2.8 Sensitivity Analysis

For this study, a sensitivity analysis was performed on the subset of patients who presented with STEMI. Additionally, a sensitivity analysis was conducted to evaluate the robustness of the SMOTE method by comparing it with the Adaptive Synthetic Sampling (ADASYN) technique, which generates synthetic samples weighted based on data density to address class imbalance. Hyperparameter tuning was conducted for the top‐performing model using the grid search method. All statistical and model development details were the same for these sensitivity analyses.

### 2.9 Ethical Considerations

Institutional Review Board (IRB) and Ethics Committee at THC approved this study (IR.TUMS.THC.REC.1399.045). This study was retrospective, utilizing deidentified data from THC's electronic registry, so an informed consent waiver was granted by the IRB.

## 3 Results

### 3.1 Patient Characteristics

The study cohort comprised 7422 patients hospitalized for AMI. Of these, 129 patients (1.74%) experienced in‐hospital mortality, while 7293 survived. The mean age of the cohort was 62.03 ± 11.49 years, with a significantly higher age observed in patients who experienced in‐hospital mortality (70.92 ± 11.64 years) compared to those who survived (61.88 ± 11.43 years) (*p* < 0.001).

Males constituted 76.2% of the total cohort, with a significantly lower proportion among those who experienced in‐hospital mortality (65.89%) compared to survivors (76.42%) (*p* = 0.007). Patients who died were significantly older, with a mean age of 70.92 ± 11.64 years compared to 61.88 ± 11.43 years for survivors (*p* < 0.001). Additionally, patients who died had significantly lower LVEF, averaging 33.24 ± 10.59% compared to 43.46 ± 8.07% in survivors (*p* < 0.001). Significant differences were also found in total cholesterol levels (149.48 ± 43.37 mg/dL in nonsurvivors vs. 161.26 ± 41.51 mg/dL in survivors, *p* = 0.003), triglycerides (121.51 ± 67.94 mg/dL vs. 152.29 ± 104.97 mg/dL, *p* < 0.001), LDL‐C (94.06 ± 35.75 mg/dL vs. 102.98 ± 34.66 mg/dL, *p* = 0.006), and fasting blood sugar (190.38 ± 111.08 mg/dL vs. 132.29 ±59.55 mg/dL, *p* < 0.001).

Medical history also revealed significant differences between the two groups. Patients who died had higher rates of diabetes (58.91% vs. 39.35%, *p* < 0.001), heart failure with reduced ejection fraction (17.83% vs. 2.48%, *p* < 0.001), chronic kidney disease (11.63% vs. 1.38%, *p* < 0.001), previous stroke (10.08% vs. 2.93%, *p* < 0.001), and left main coronary artery disease (11.63% vs. 2.61%, *p* < 0.001). A greater prevalence of three‐vessel coronary artery disease was also noted among nonsurvivors (58.14% vs. 30.36%, *p* < 0.001).

On the other hand, factors that did not differ significantly between the two groups included body mass index (BMI), where the overall cohort had a mean BMI of 28.04 ± 4.44 kg/m^2^, with no significant difference between nonsurvivors (27.77 ± 4.1 kg/m^2^) and survivors (28.05 ± 4.45 kg/m^2^) (*p* = 0.456). Similarly, waist circumference (99.22 ± 9.63 cm in nonsurvivors vs. 99.76 ± 10.46 cm in survivors, *p* = 0.534) and HDL‐C levels (38.61 ± 10.64 mg/dL vs. 38.59 ± 9.51 mg/dL, *p* = 0.99) showed no significant differences. Additional factors, including smoking status (*p* = 0.07), lesion length (*p* = 0.823), and opium use (*p* = 0.509), were also not significantly different between the groups. Table 1 details all baseline characteristics, including lesion type, coronary artery disease extension, and TIMI flow scores pre‐ and post‐PCI.

**Table 1:** Baseline characteristics of patients with AMI.

Variable	Total cohort	In‐hospital mortality	No in‐hospital mortality	*p* value
Number of patients	7422	129	7293	
LVEF	43.28 ± 8.23	33.24 ± 10.59	43.46 ± 8.07	*p* < 0.001
Age (years)	62.03 ± 11.49	70.92 ± 11.64	61.88 ± 11.43	*p* < 0.001
BMI	28.04 ± 4.44	27.77 ± 4.1	28.05 ± 4.45	0.456
Waist circumference (cm)	99.74 ± 10.44	99.22 ± 9.63	99.76 ± 10.46	0.534
Total cholesterol (mg/dL)	161.05 ± 41.56	149.48 ± 43.37	161.26 ± 41.51	0.003
Triglycerides (mg/dL)	151.75 ± 104.51	121.51 ± 67.94	152.29 ± 104.97	*p* < 0.001
LDL‐C (mg/dL)	102.82 ± 34.69	94.06 ± 35.75	102.98 ± 34.66	0.006
HDL‐C (mg/dL)	38.59 ± 9.53	38.61 ± 10.64	38.59 ± 9.51	0.99
FBS (mg/dL)	133.29 ± 61.28	190.38 ± 111.08	132.29 ± 59.55	*p* < 0.001
Creatinine (mg/dL)	0.98 ± 0.5	1.35 ± 0.76	0.98 ± 0.5	*p* < 0.001
Hemoglobin (g/dL)	14.91 ± 1.85	13.86 ± 2.4	14.93 ± 1.84	*p* < 0.001
Pre‐PCI stenosis (%)	94.86 ± 8.33	98.64 ± 3.83	94.8 ± 8.38	*p* < 0.001
Post‐PCI stenosis (%)	3.99 ± 16.84	17.83 ± 34.98	3.75 ± 16.25	*p* < 0.001
Lesion length (mm)	27.13 ± 13.14	26.89 ± 12.47	27.14 ± 13.16	0.823
Stent diameter (mm)	3 ± 0.46	2.89 ± 0.41	3.01 ± 0.46	0.002
Contrast volume (mL)	225.35 ± 64.02	224.73 ± 77.82	225.37 ± 63.76	0.926
History of STEMI	4.5% (*n* = 332)	6.2% (*n* = 8)	4.44% (*n* = 324)	0.457
History of NSTEMI	1.5% (*n* = 111)	3.1% (*n* = 4)	1.47% (*n* = 107)	0.25
History of unstable angina	1.1% (*n* = 82)	0.78% (*n* = 1)	1.11% (*n* = 81)	0.99
History of chronic coronary syndrome	0.4% (*n* = 33)	0% (*n* = 0)	0.45% (*n* = 33)	0.921
Sex (male)	76.2% (*n* = 5658)	65.89% (*n* = 85)	76.42% (*n* = 5573)	0.007
Family history of CAD	18.8% (*n* = 1395)	10.08% (*n* = 13)	18.95% (*n* = 1382)	0.014
Dyslipidemia	56.9% (*n* = 4221)	39.53% (*n* = 51)	57.18% (*n* = 4170)	*p* < 0.001
Diabetes mellitus	39.7% (*n* = 2946)	58.91% (*n* = 76)	39.35% (*n* = 2870)	*p* < 0.001
Hypertension	49.1% (*n* = 3644)	48.84% (*n* = 63)	49.1% (*n* = 3581)	0.9
Smoking	45.4% (*n* = 3372)	37.21% (*n* = 48)	45.58% (*n* = 3324)	0.07
Opium use	16.5% (*n* = 1223)	13.95% (*n* = 18)	16.52% (*n* = 1205)	0.509
History of HFrEF	2.7% (*n* = 204)	17.83% (*n* = 23)	2.48% (*n* = 181)	*p* < 0.001
History of VHD	1.7% (*n* = 127)	8.53% (*n* = 11)	1.59% (*n* = 116)	*p* < 0.001
History of CVA	3.1% (*n* = 227)	10.08% (*n* = 13)	2.93% (*n* = 214)	*p* < 0.001
History of lung disease	2.2% (*n* = 164)	3.1% (*n* = 4)	2.19% (*n* = 160)	0.694
History of ESRD	1.6% (*n* = 116)	11.63% (*n* = 15)	1.38% (*n* = 101)	*p* < 0.001
Previous CABG	7.1% (*n* = 523)	3.1% (*n* = 4)	7.12% (*n* = 519)	0.111
Previous PCI	12.2% (*n* = 908)	13.18% (*n* = 17)	12.23% (*n* = 892)	0.849
Previous shock	0.3% (*n* = 27)	9.3% (*n* = 12)	0.21% (*n* = 15)	*p* < 0.001
Left main coronary artery lesion	2.7% (*n* = 205)	11.63% (*n* = 15)	2.61% (*n* = 190)	*p* < 0.001
Pre‐PCI TIMI flow				
TIMI 0	43.4% (*n* = 3211)	69.77% (*n* = 90)	42.79% (*n* = 3121)	*p* < 0.001
TIMI 1	5.2% (*n* = 392)	10.08% (*n* = 13)	5.2% (*n* = 379)	0.023
TIMI 2	17.6% (*n* = 1308)	11.63% (*n* = 15)	17.73% (*n* = 1293)	0.09
TIMI 3	33.8% (2511)	8.53% (*n* = 11)	34.28% (*n* = 2500)	*p* < 0.001
Post‐PCI TIMI flow				
TIMI 0	1.8% (*n* = 133)	14.73% (*n* = 19)	1.56% (*n* = 114)	*p* < 0.001
TIMI 1	0.5% (*n* = 36)	2.33% (*n* = 3)	0.45% (*n* = 33)	0.016
TIMI 2	3.2% (*n* = 240)	14.73% (*n* = 19)	3.03% (*n* = 221)	*p* < 0.001
TIMI 3	94.5% (*n* = 7013)	68.22% (*n* = 88)	94.95% (*n* = 6925)	*p* < 0.001
CAD extension				
SVD	34.3% (*n* = 2549)	23.26% (*n* = 30)	34.54% (*n* = 2519)	*p* < 0.001
2VD	34.6% (*n* = 2571)	18.6% (*n* = 24)	34.92% (*n* = 2547)	*p* < 0.001
3VD	30.9% (*n* = 2289)	58.14% (*n* = 75)	30.36% (*n* = 2214)	*p* < 0.001
Lesion type (ACC/AHA classification)				
Type A	0.16% (*n* = 12)	0% (*n* = 0)	0.16% (*n* = 12)	0.99
Type B	11.8% (*n* = 876)	4.65% (*n* = 6)	11.93% (*n* = 870)	0.016
Type C	14.9% (*n* = 1107)	10.08% (*n* = 13)	15% (*n* = 1094)	0.152
Type D	73.1% (*n* = 5427)	85.27% (*n* = 110)	72.91% (*n* = 5317)	0.002

*Note:* Data were expressed as mean ± SD or as percent (number).

Abbreviations: ACC/AHA, American College of Cardiology/American Heart Association; AMI, acute myocardial infarction; BMI, body mass index; CABG, coronary artery bypass graft; CAD, coronary artery disease; CVA, cerebrovascular accident; ESRD, end‐stage renal disease; FBS, fasting blood sugar; HDL‐C, high‐density lipoprotein cholesterol; HFrEF, heart failure with reduced ejection fraction; LDL‐C, low‐density lipoprotein cholesterol; LVEF, left ventricular ejection fraction; NSTEMI, non‐ST‐elevation myocardial infarction; PCI, percutaneous coronary intervention; Post‐PCI stenosis, post‐percutaneous coronary intervention stenosis; Pre‐PCI stenosis, pre‐percutaneous coronary intervention stenosis; STEMI, ST‐elevation myocardial infarction; TIMI, Thrombolysis in Myocardial Infarction; TIMI 0, no perfusion (complete occlusion); TIMI 1, penetration without perfusion (minimal flow); TIMI 2, partial perfusion (sluggish but present flow); TIMI 3, complete perfusion (normal flow); SVD, single‐vessel disease; 2VD, two‐vessel disease; 3VD, three‐vessel disease; VHD, valvular heart disease.

### 3.2 Model Evaluation

**Figure 1:**
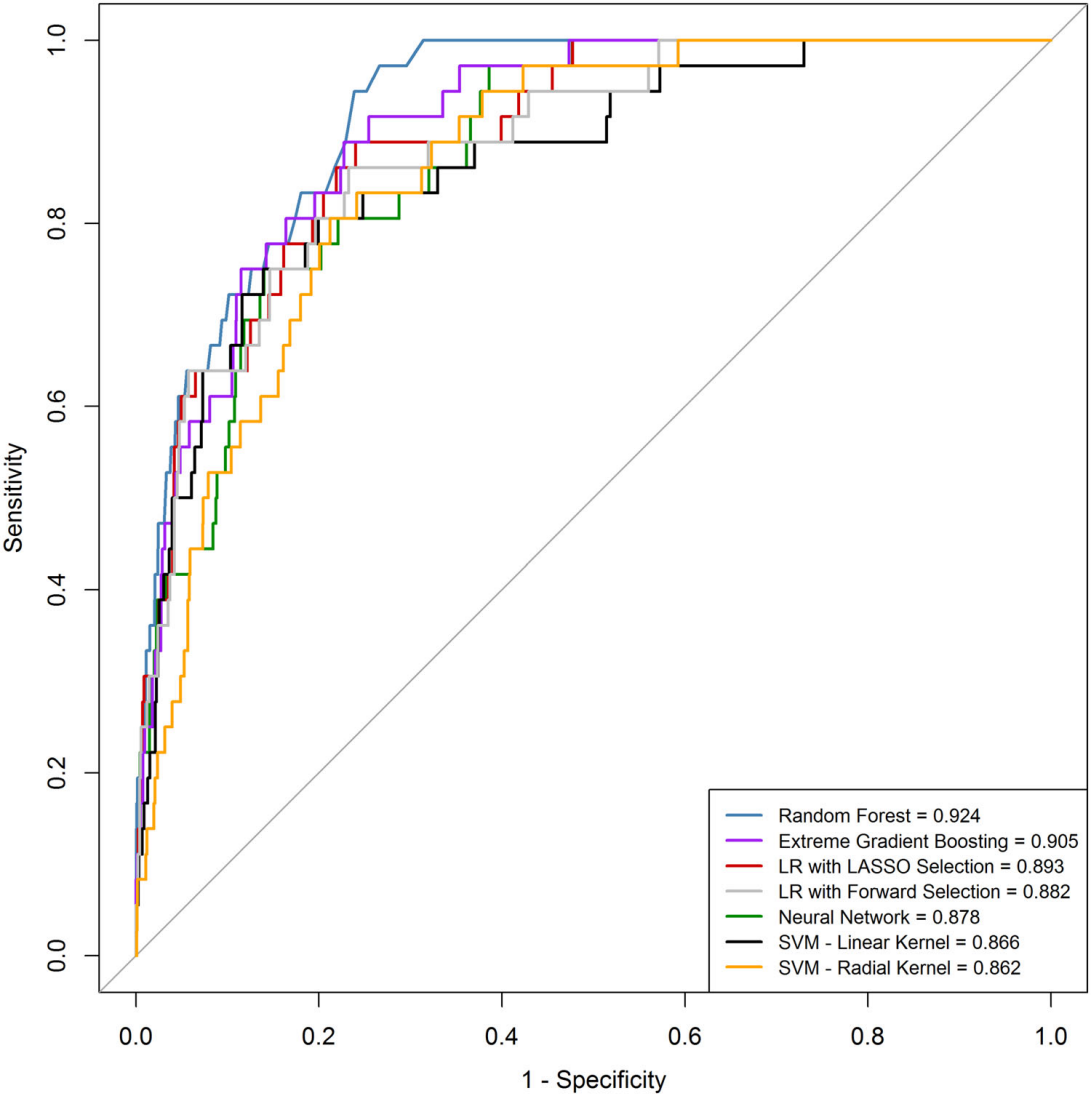
Displaying the ROC curves comparing the performance of various models (AUC values shown in the figure). This figure displays the receiver operating characteristic (ROC) curves for several machine learning models predicting in‐hospital mortality in patients with AMI. The models evaluated include random forest, logistic regression (LR) with LASSO selection, eXtreme Gradient Boosting (XGBoost), neural network, and support vector machines (SVM) with both linear and radial kernels, as well as logistic regression with forward selection. Random forest demonstrated the highest area under the curve (AUC = 0.924), indicating superior predictive performance. The AUC values for the other models were as follows: LR with LASSO selection (AUC = 0.893), XGBoost (AUC = 0.905), neural network (AUC = 0.878), SVM with the linear kernel (AUC = 0.866), SVM with the radial kernel (AUC = 0.862), and LR with forward selection (AUC = 0.882). The ROC curves illustrate each model's sensitivity versus 1‐specificity, with the diagonal line representing a random classifier. AMI, acute myocardial infarction; AUC, area under the curve; LR, logistic regression; ROC, receiver operating characteristic; SVM, support vector machines; XGBoost, eXtreme Gradient Boosting.

Figure 1 presents the AUC‐ROC curves for the models evaluated in this study. RF demonstrated the highest performance with an AUC of 0.924 (95% CI 0.893–0.954), followed by XGBoost (AUC 0.905, 95% CI 0.866–0.943) and logistic regression with LASSO selection (AUC 0.893, 95% CI 0.848–0.939). Logistic regression with forward selection achieved an AUC of 0.882 (95% CI 0.830–0.934), while the NN model recorded an AUC of 0.878 (95% CI 0.832–0.932). SVM with a linear kernel had an AUC of 0.866 (95% CI 0.805–0.927), and the radial kernel variant of SVM produced an AUC of 0.862 (95% CI 0.815–0.909). These results highlight the strong performance of ensemble‐based models such as RF and XGBoost for predicting in‐hospital mortality among AMI patients.

Additionally, Figure S1 shows the performance of the models without applying SMOTE. Under these conditions, the RF and NN models achieved the highest AUC (0.905 each), followed by logistic regression with LASSO selection (AUC = 0.900) and logistic regression with forward selection (AUC = 0.893). XGBoost had an AUC of 0.884, while SVM with a radial kernel achieved an AUC of 0.848. SVM with a linear kernel demonstrated the lowest performance, with an AUC of 0.732. These results indicate that while SMOTE slightly improved the AUC for some models, the reduction in performance without SMOTE was minimal, maintaining overall model reliability. The details of the performance comparison of ML models are available in Table S2.

The mixed model, combining the GRACE score with the top 5 predictors identified in this study (LVEF, fasting blood glucose (FBS), serum creatinine (CR), age, and hemoglobin (Hb)), was evaluated across multiple ML approaches. Logistic regression with forward selection achieved an AUC of 0.872, while logistic regression with LASSO selection recorded an AUC of 0.871. RF achieved an AUC of 0.853, and XGBoost had an AUC of 0.834. The NN model achieved an AUC of 0.800, while SVM with linear and radial kernels showed the lowest performance, with AUCs of 0.721 and 0.711, respectively. The corresponding ROC curves for these models are presented in Figure S2, and the details of the performance comparison of ML models are available in Table S3.

Table 2 summarizes the key metrics, including each model's sensitivity, specificity, Brier score, and F1 score. RF achieved the highest sensitivity (97%) and the lowest Brier score (0.014), along with the highest F1 score (0.992). Logistic regression with LASSO selection also performed well, with a sensitivity of 89%, specificity of 76%, a Brier score of 0.016, and an F1 score of 0.99. XGBoost followed closely, with a sensitivity of 92%, specificity of 75%, a Brier score of 0.015, and an F1 score of 0.991.

**Table 2:** Performance comparison of machine learning models for in‐hospital mortality prediction.

Model Name	AUC (95% CI)	Sensitivity	Specificity	Brier score	F1 score
Logistic regression with forward selection	0.882 (0.830–0.934)	0.86	0.77	0.017	0.99
Logistic regression with LASSO selection	0.893 (0.848–0.939)	0.89	0.76	0.016	0.99
Neural network	0.878 (0.832–0.932)	0.75	0.86	0.018	0.988
Random forest	0.924 (0.893–0.954)	0.97	0.73	0.014	0.992
eXtreme Gradient Boosting	0.905 (0.866–0.943)	0.92	0.75	0.015	0.991
SVM—linear kernel	0.866 (0.805–0.927)	0.75	0.86	0.017	0.992
SVM—radial kernel	0.862 (0.815–0.909)	0.81	0.79	0.018	0.989

Abbreviations: AUC, area under the curve; CI, confidence interval; SVM, support vector machine.

The NN exhibited high specificity (86%) and an F1 score of 0.988, although it had a lower sensitivity (75%). SVM with a linear kernel shared a similar pattern, with a sensitivity of 75%, specificity of 86%, and an F1 score of 0.992. SVM with a radial kernel demonstrated balanced performance, with a sensitivity of 81%, specificity of 79%, a Brier score of 0.018, and an F1 score of 0.989. Logistic regression with forward selection showed a sensitivity of 86%, specificity of 77%, a Brier score of 0.017, and an F1 score of 0.99. The comparison of all ML models across different settings (the main analysis, the analysis without SMOTE, and the mixed model) is provided in Table S4.

**Figure 2:**
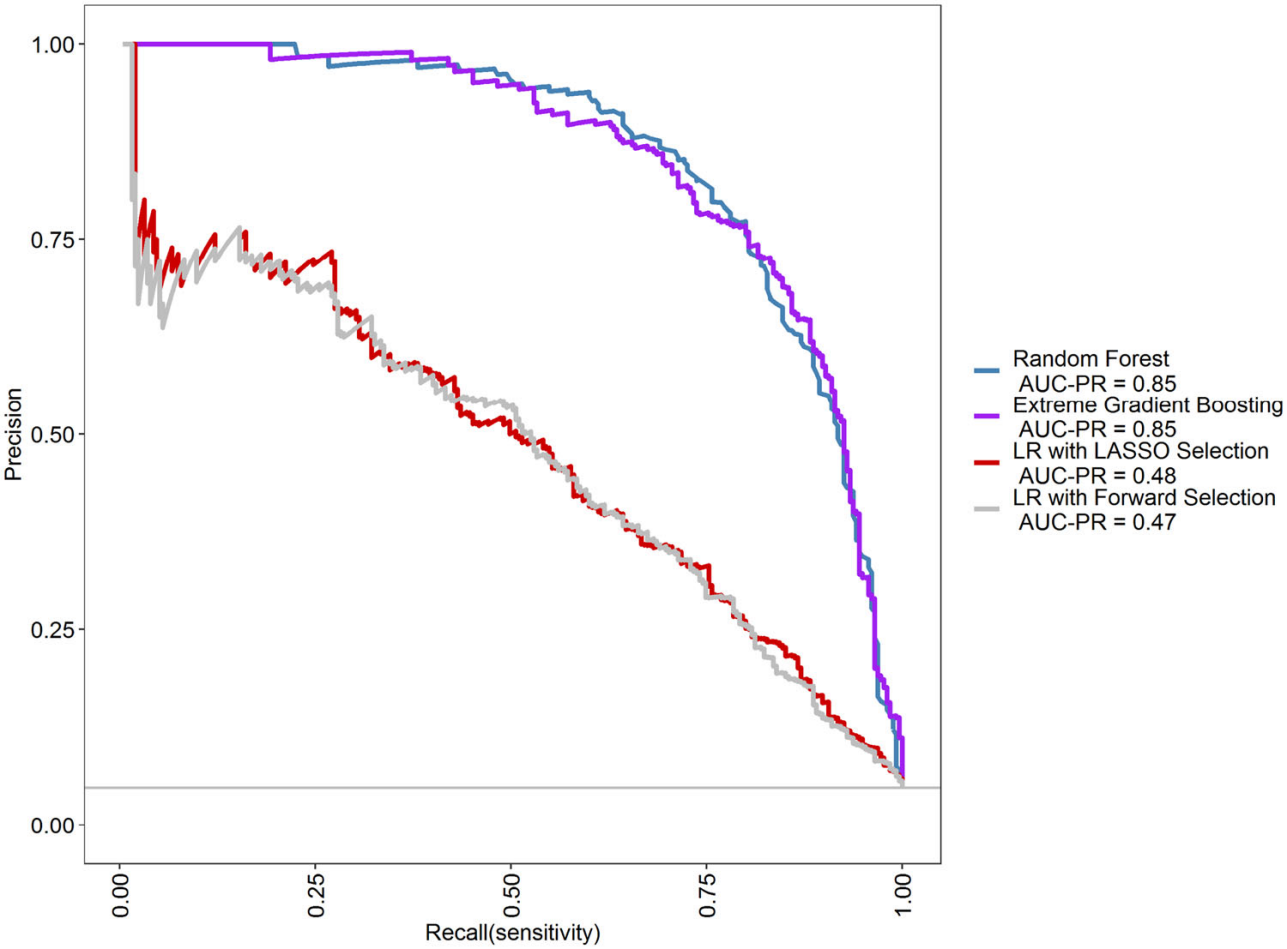
Precision‐recall curves of various machine learning models (AUC‐PR values are shown in the figure). This figure displays the PR curves for several machine‐learning models used to predict in‐hospital mortality among AMI patients. The models include random forest, eXtreme Gradient Boosting (XGBoost), neural network, and logistic regression (LR) with LASSO selection. The random forest model demonstrated the highest precision‐recall area under the curve (AUC‐PR = 0.85), closely followed by XGBoost (AUC‐PR = 0.85). The LR with the LASSO selection model had an AUC‐PR of 0.48, while the LR with forward selection showed the lowest precision‐recall performance (AUC‐PR = 0.47). Higher AUC‐PR values indicate better performance, reflecting the model's ability to maintain a balance between precision and recall, which is crucial for identifying true positive cases and minimizing false positives in imbalanced data sets. AMI, acute myocardial infarction; AUC‐PR, area under the precision‐recall curve; LR, logistic regression; LASSO, Least Absolute Shrinkage and Selection Operator; PR, precision‐recall; XGBoost, eXtreme Gradient Boosting.

Figure 2 presents the PRC for the top models, with RF and XGBoost showing the highest AUC‐PR (0.85 each). Logistic regression with LASSO selection demonstrated a moderate AUC‐PR of 0.48, while logistic regression with forward selection had the lowest precision‐recall performance with an AUC‐PR of 0.47. RF and XGBoost maintained high precision across a wide range of recall values, indicating strong performance in identifying true positives. A high AUC‐PR reflects the model's ability to balance precision and recall, ensuring fewer false positives while accurately capturing true positive cases, which is particularly crucial in imbalanced data sets. For the mixed model, RF achieved an AUC‐PR of 0.76, followed by XGBoost with an AUC‐PR of 0.73. Both logistic regression with LASSO selection and logistic regression with forward selection recorded an AUC‐PR of 0.47 (Figure S3)

Figure S4 shows the calibration plot for the main analysis, while Figure S5 presents the calibration plots for the mixed model setting. These plots compare the predicted probabilities with observed outcomes across different models, providing insights into the models' calibration performance.

### 3.3 Feature Importance Evaluation

The correlation heatmap between variables is shown in Figure S6. As depicted, high correlations were observed between LDL‐C and total cholesterol, as well as between BMI and waist circumference. These relationships highlight potential interactions between variables, which may influence the model's performance.

Figure S7 presents the SHAP beeswarm plot, highlighting the most important predictors of in‐hospital mortality in the XGBoost model. LVEF, FBS, and serum CR ranked as the top predictors, followed by pre‐PCI stenosis, contrast volume, and dyslipidemia. Additional influential predictors included age, CAD extension, stent diameter, and Hb. The plot illustrates how each feature influences the model's predictions, with higher feature values (yellow) generally associated with increased mortality risk, while lower feature values (purple) correspond to reduced risk. The horizontal position of each dot indicates the SHAP value, representing the impact of the feature on the prediction, while the density of dots demonstrates the distribution of feature effects across patients.

Figures S8.1–S8.8 display the SHAP dependence plots for key variables such as age, BMI, serum CR, FBS, LDL‐C, LVEF, total cholesterol, and waist circumference based on XGBoost. These plots show the specific relationships between each feature and the SHAP values. Age and LVEF demonstrated significant associations with in‐hospital mortality, with U‐shaped and inverse relationships, respectively. Higher age and lower LVEF were strongly associated with increased mortality risk.

**Figure 3:**
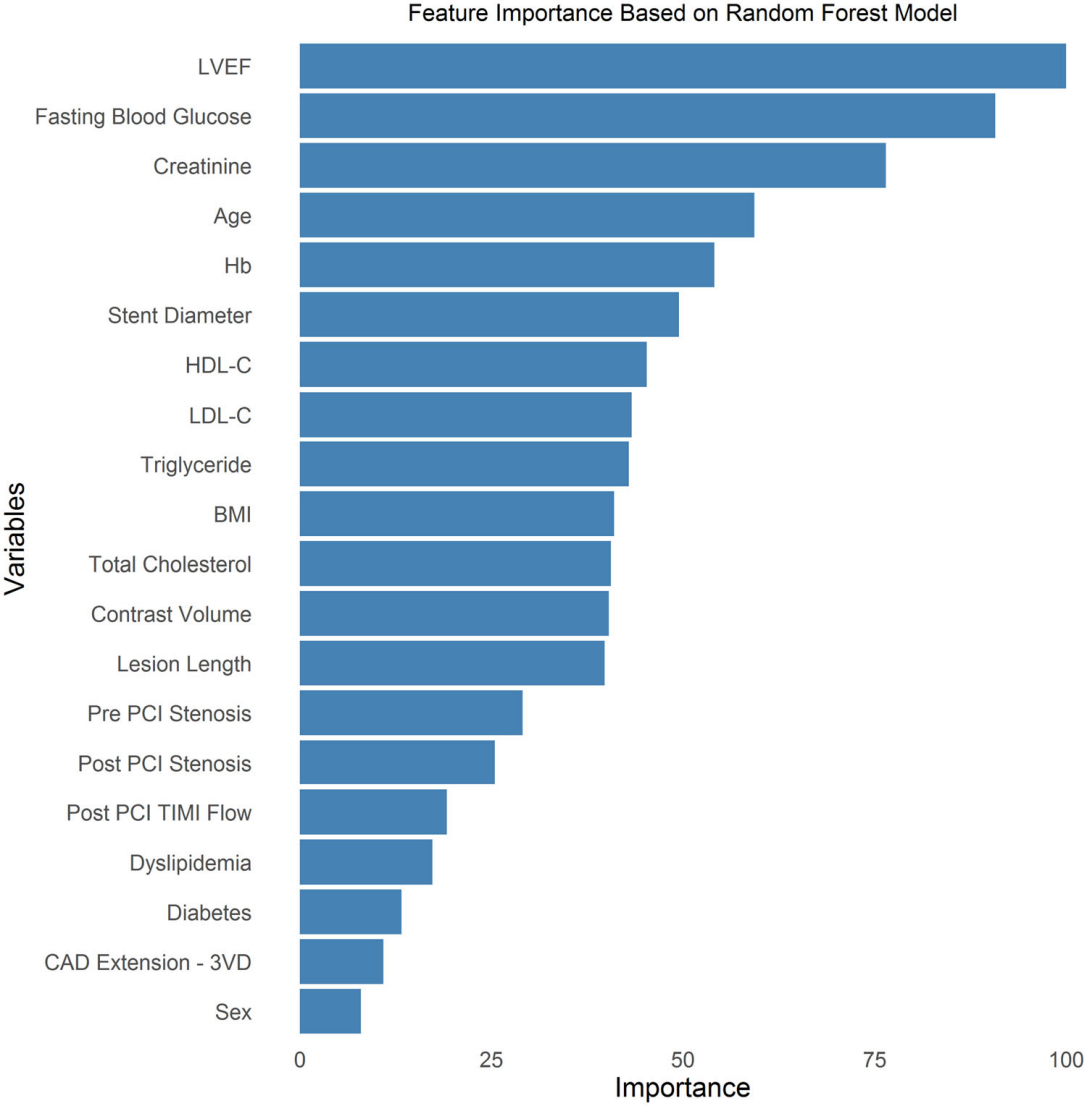
Feature importance of variables based on the random forest model. This bar chart displays the ranked importance of variables in the random forest model for predicting in‐hospital mortality among AMI patients. The most influential predictors are LVEF, fasting blood glucose, and serum creatinine, followed by age, hemoglobin, and stent diameter. Other significant contributors include HDL‐C, LDL‐C, triglycerides, BMI, total cholesterol, and contrast volume. Additional variables such as lesion length, pre‐PCI stenosis, post‐PCI stenosis, post‐PCI TIMI flow, dyslipidemia, diabetes, CAD extension (three‐vessel disease), and sex also demonstrate notable importance. The importance of each variable is quantified based on its contribution to the model's predictive performance, with higher values indicating greater influence. AMI, acute myocardial infarction; BMI, body mass index; CAD, coronary artery disease; HDL‐C, high‐density lipoprotein cholesterol; LDL‐C, low‐density lipoprotein cholesterol; LVEF, left ventricular ejection fraction; PCI, percutaneous coronary intervention; TIMI, Thrombolysis in Myocardial Infarction.

Figure 3 illustrates the feature importance rankings based on the RF model. LVEF, FBS, and CR were the top predictors, followed by age and Hb. Other relevant predictors included stent diameter, HDL‐C, LDL‐C, triglyceride, BMI, total cholesterol, contrast volume, lesion length, and pre‐PCI stenosis.

### 3.4 Sensitivity Analysis

A sensitivity analysis was performed on a subset of patients who presented with STEMI to assess the robustness of the model's performance. Figure S9 presents the AUC‐ROC curves for the sensitivity analysis. RF maintained a strong performance with an AUC of 0.900, slightly reduced compared to the main analysis (AUC = 0.924). XGBoost achieved a comparable AUC of 0.901, almost identical to the main analysis (AUC = 0.905). Logistic regression with LASSO selection showed a marginal decrease in performance, with an AUC of 0.891 compared to 0.893 in the main analysis. Logistic regression with forward selection remained consistent with an AUC of 0.882 in both analyses. The NN showed a more notable reduction in performance, with an AUC of 0.854 compared to 0.878 in the main analysis. SVM with a radial kernel had an AUC of 0.830, slightly lower than its main analysis counterpart (AUC = 0.862). SVM with a linear kernel showed the most significant decrease, dropping to an AUC of 0.670 from 0.866 in the main analysis. Despite these changes, most models retained their ability to discriminate between patients with and without in‐hospital mortality, showcasing robust performance in this subset of patients. Table S5 provides the sensitivity, specificity, Brier score, and F1 score for each model in this sensitivity analysis.

The feature importance rankings (Figure S10), as determined by the RF model in the sensitivity analysis, identified LVEF, FBS, and CR as the top predictors, consistent with the primary analysis. Hb and age also emerged as significant predictors, with Hb ranking higher than in the main analysis. Predictors such as stent diameter, lesion length, and post‐PCI stenosis exhibited slight changes in importance. Additional features like history of HFrEF and CAD extension—2VD appeared as moderately important in the sensitivity analysis. Overall, the rankings remained relatively stable, reinforcing the robustness of key predictors even when focusing on the STEMI subgroup.

When examining the precision‐recall performance (Figure S11), the AUC‐PR showed a decrease across all models in the sensitivity analysis. RF maintained the highest AUC‐PR at 0.75, followed by XGBoost at 0.65. Logistic regression with LASSO selection exhibited a notable drop with an AUC‐PR of 0.30, while logistic regression with forward selection demonstrated the lowest precision‐recall performance with an AUC‐PR of 0.29. These results suggest a decline in precision for logistic regression models under the sensitivity analysis, while RF and XGBoost showed relatively stable performance.

In the sensitivity analysis using ADASYN for class imbalance handling on the main population (Figure S12), the RF model demonstrated the highest AUC‐ROC of 0.887, indicating strong performance, though slightly reduced compared to the AUC‐ROC of 0.924 achieved with SMOTE. XGBoost followed with an AUC‐ROC of 0.852, slightly lower than its performance with SMOTE (AUC‐ROC = 0.905). Logistic regression with LASSO selection achieved an AUC‐ROC of 0.844, showing a modest decline from its SMOTE‐based performance (AUC‐ROC = 0.893). Similarly, logistic regression with forward selection and the SVM linear kernel exhibited minor reductions, with AUC‐ROC values of 0.839 and 0.841 under ADASYN compared to 0.882 and 0.866, respectively, with SMOTE. The NN model saw a slight decrease (AUC‐ROC = 0.822 with ADASYN vs. 0.878 with SMOTE). SVM with a radial kernel showed the most notable drop in performance, with an AUC‐ROC of 0.752 compared to 0.862 under SMOTE. Detailed metrics for sensitivity, specificity, F1 score, and Brier score are presented in Table S6. Moreover, the missing data for each variable and the optimal hyperparameters for RF, XGBoost, and SVM are available in Tables S7 and S8, respectively.

Hyperparameter tuning was performed for the RF model using a grid search method to optimize performance. The process involved evaluating different values for key parameters, such as the number of predictors randomly selected at each split (mtry) and the number of trees (ntree). Figure S13 illustrates the relationship between mtry and cross‐validated ROC, highlighting a decreasing trend in performance as the number of predictors increases. Similarly, Figure S14 displays the variation in cross‐validation accuracy with changes in ntree, identifying the optimal configuration at ~6000 trees for maximum accuracy.

## 4 Discussion

### 4.1 Key Findings and Model Performance

Our study used ML models to predict in‐hospital mortality among AMI patients. The RF model achieved the highest AUC of 0.900 (95% CI: 0.864–0.937), with LVEF, FBS, and CR identified as the top predictors. Logistic regression with LASSO selection and SVM also performed well, with AUCs of 0.865 and 0.875, respectively. Sensitivity analysis confirmed the robustness of these results, with only minor reductions in AUC observed when SMOTE was not applied (RF AUC = 0.884). These findings underscore the significance of key clinical and biochemical features in predicting mortality for AMI patients.

### 4.2 Research on ML in AMI Prognosis

ML has rapidly become a transformative tool for predicting in‐hospital mortality in AMI patients, offering significant advantages over traditional statistical models. Unlike conventional methods, which struggle with complex, high‐dimensional data and nonlinear relationships among clinical features, ML models excel in these areas, providing more accurate and personalized predictions. This capability is precious for high‐risk scenarios such as STEMI and NSTEMI.

A study by Lee and colleagues on AMI patients in Korea [45] exemplifies ML's potential in AMI prognosis, comparing the performance of supervised and unsupervised models, including logistic regression with regularization, RF, SVM, and XGBoost, against traditional models in NSTEMI patients. ML models demonstrated superior performance compared to traditional approaches, particularly in predicting in‐hospital mortality, achieving an AUC of 0.889 compared to 0.873 for traditional models. ML models also maintained strong predictive power for long‐term mortality, with AUCs of 0.849 and 0.860 for 3‐month and 12‐month mortality, respectively. This highlights ML's ability to detect subtle patterns in patient data that traditional models might miss, making them particularly effective for heterogeneous clinical profiles like NSTEMI.

Similarly, a 5‐year cohort in China by Zhao et al. [46] focused on STEMI patients, using several ML algorithms trained on data sets containing pre‐reperfusion therapy variables. The SVM model performed best, with an AUC of 0.919, an accuracy of 85.62%, and a G‐mean of 84.93%. Notably, the SVM model also performed well with a simplified data set, demonstrating its clinical utility for rapid decision‐making with limited data. These findings are aligned with the results of our study; however, while we found the SVM model to be effective (AUC = 0.875), the RF model achieved the highest AUC of 0.900 among all models.

### 4.3 Translating ML Insights Into Clinical Practice

The success of ML models in predicting in‐hospital mortality for AMI is largely due to their ability to integrate diverse patient data, including clinical features, comorbidities, laboratory results, and demographic factors. Khera et al. [47] demonstrated this with data from the American College of Cardiology (ACC) Chest Pain‐MI Registry, which included over 755 000 AMI patients, in a nationwide registry. Their study compared XGBoost and a NN with traditional logistic regression models, finding that XGBoost demonstrated a marginally higher predictive accuracy compared to logistic regression (C‐statistic of 0.90 vs. 0.89). XGBoost also excelled in risk classification, reclassifying 27% of moderate‐to‐high‐risk patients as low‐risk, aligning more closely with actual outcomes. The study emphasized the importance of age, previous AMI, and HF severity (Killip class) in mortality predictions. Feature importance analysis revealed that age was a dominant factor, with its effect modulated by HF severity and kidney function. This demonstrates ML models' ability to capture complex, nonlinear dependencies among variables.

In comparison to our study, the SHAP plot for RF identified LVEF, serum CR, and FBS as top predictors of in‐hospital mortality, with age, pre‐PCI stenosis, and CAD extension also significant. SHAP dependence plots showed that higher age and lower LVEF were strongly associated with increased mortality risk, mirroring patterns observed in Khera and colleagues's study [47]. However, our NN model underperformed compared to XGBoost and RF (AUC 0.790 vs. 0.900 for RF), though it remained competitive without oversampling techniques (AUC 0.897).

Recent research also highlights the importance of integrating novel biomarkers, such as brachial pre‐ejection period (bPEP) and ejection time (bET), into ML models, which significantly improved long‐term mortality predictions [48]. These findings underscore the need to integrate novel variables identified by ML models into clinical decision‐making, demonstrating their potential as valuable tools compared to traditional risk‐scoring systems.

The effectiveness of ML models in predicting in‐hospital mortality for AMI can be attributed to their ability to incorporate a broad range of patient data, including crucial clinical variables. Lee et al. [45] found that even moderate increases in CR significantly raise mortality risks, especially in patients with pre‐existing CKD. Moreover, their findings highlighted the significance of pre‐PCI stenosis and CAD extension as predictors of in‐hospital mortality, which was also confirmed by our study's XGBoost and RF models.

Additionally, SHAP analysis by Lee et al. [45] revealed a U‐shaped relationship between LVEF and mortality, with both very low and very high values critical. Reduced LVEF (< 40%) is a strong mortality predictor, indicating systolic dysfunction and a heightened risk of complications such as cardiogenic shock and ventricular arrhythmias. These findings corroborate our study's results, confirming the critical role of LVEF and CR in predicting AMI outcomes, as the second and third most important features, respectively.

Another important aspect of ML models is their ability to optimize performance with fewer variables. Goriki et al. [49] developed a laboratory‐based risk score for STEMI patients, identifying predictors such as estimated glomerular filtration rate (eGFR < 45 mL/min/1.73 m^2^), platelet count (< 150 000/μL), albumin (≤ 3.5 g/dL), high‐sensitivity troponin I (> 1.6 ng/mL), and blood sugar (≥ 200 mg/dL). This model achieved AUCs of 0.853 in the derivation cohort and 0.879 in the validation cohort, demonstrating the feasibility of using simplified, rapid assessments in emergency settings without sacrificing predictive accuracy. This is in parallel to our results that laboratory assessments such as total cholesterol and LDL‐C, along with the total triglyceride, HDL, and Hb showed significant differences between survivors and nonsurvivors (*p* = 0).

Hyperglycemia at admission emerged as a strong predictor in our study, reflecting metabolic stress and inflammation, exacerbating myocardial injury, and delaying recovery. From a pathophysiological view, it promotes a prothrombotic environment and impairs the immune response [50]. SHAP analysis highlighted that acute hyperglycemia, even in nondiabetic patients, substantially increases the risk of complications such as HF and atrial fibrillation [51]. This arrhythmia which is often observed in AMI patients, further elevates mortality risks by impairing cardiac output and inducing arrhythmic and ischemic stress [45].

The integration of ML models with traditional cardiology risk scores, such as the GRACE, TIMI scores, and Acute Coronary Treatment and Intervention Outcomes Network—Get With The Guidelines (ACTION‐GWTG), has also been explored [45, 52]. Shouval et al. [52] compared various ML algorithms with these established risk scores in predicting 30‐day mortality in STEMI patients. The study, involving over 2700 patients, found that the best‐performing ML models achieved AUCs between 0.87 and 0.91, comparable to or exceeding the GRACE score's AUC of 0.87.

Another similar observational study on ~15 000 AMI patients revealed that for in‐hospital mortality, RF and XGBoost had the highest AUCs of 0.889 and 0.888, respectively, outperforming the TIMI score (AUC: 0.669) but showed comparable performance to the modified ACTION‐GWTG (AUC: 0.884). In predicting 3‐month mortality, LASSO and elastic net regressions led with an AUC of 0.849, surpassing GRACE (AUC: 0.777) and ACTION‐GWTG (AUC: 0.795). For 12‐month mortality, most ML models, excluding SVM, maintained an AUC > 0.8, contrasting with TIMI's AUC of 0.675 and ACTION‐GWTG's AUC of 0.790. Notably, LASSO, Ridge, and elastic net regressions, along with XGBoost, achieved significantly higher AUCs compared to traditional models [45]. In our study, the mixed model, combining the GRACE score with top predictors (LVEF, age, FBS, serum CR, and Hb), achieved competitive performance across all methods, with RF maintaining an AUC‐ROC of 0.853 and LASSO regression recording an AUC‐ROC of 0.871.

In addition, studies have shown that patients with more extensive coronary blockages (e.g., three‐vessel disease (3VD)) face higher in‐hospital mortality rates due to increased ischemic burden. A pooled analysis of angiographic data on 3032 patients with AMI undergoing PCI showed that patients with multivessel disease have worse outcomes compared to those with limited disease [53]. This aligns with Lee and colleagues's finding that the survival group had a lower proportion of heart failure, cardiogenic shock, left main disease, and 3VD [45]. In line with the previous literature, our RF sensitivity analysis demonstrated 3VD and 2VD, as the least important features, with a coefficient of < 0.5 for both, though with a significant correlation.

### 4.4 Clinical Relevance of Key Predictors Threshold

Identifying precise cutoff values for LVEF, FBG, and serum CR is crucial in assessing in‐hospital mortality risk. Research indicates that lower LVEF is associated with increased mortality, particularly when it falls below 40%, where patients experience a 14% in‐hospital mortality rate compared to 5% for those with LVEF between 40% and 49%. An LVEF of 35% has been linked to higher mortality at 180 days, with a linear relationship between decreasing LVEF and mortality risk. Similarly, an LVEF ≤ 40% is a well‐established risk factor for mortality after ACS, with studies showing a higher incidence of inducible ventricular tachycardia postprimary percutaneous coronary intervention (PPCI).

Elevated FBG levels have also been associated with increased in‐hospital mortality. Patients with FBG > 110 mg/dL (6.1 mmol/L) show a higher mortality rate, while those with FBG > 144 mg/dL (8 mmol/L) in the absence of diabetes face an even greater risk. A threshold of FBG > 200 mg/dL (11.1 mmol/L) has been identified as a significant predictor of adverse hospital outcomes. Similarly, serum CR levels serve as an important marker, with levels ≥ 1.12 mg/dL (99 µmol/L) upon admission linked to increased mortality risk, and those reaching ≥ 2.0 mg/dL (177 µmol/L) during treatment associated with a high in‐hospital mortality rate. For patients with reduced LVEF, guideline‐directed medical therapy including ACE inhibitors, ARBs, beta‐blockers, MRAs, and SGLT2 inhibitors improves symptoms, prevents heart structural changes, and reduces hospitalization and sudden death risks.

Optimal glycemic control is essential for patients with elevated FBG, with lifestyle changes like weight loss, physical activity, and medical nutrition therapy reducing ASCVD risk factors.

Managing hypertension and diabetes is key to preserving kidney function, with monitoring of serum CR and potassium after starting ACE inhibitors/ARBs, MRAs, or diuretics to control cardiovascular risks.

### 4.5 Strengths, Limitations, and Future Directions

This study had some strengths. It comprised a large and diverse cohort of 7422 AMI patients from THC, ensuring robust and generalizable findings. It uses a comprehensive range of variables and advanced ML models, such as XGBoost and RF, to thoroughly evaluate predictors of in‐hospital mortality. The application of SHAP values provides clear insights into feature importance, and sensitivity analyses, including evaluations with and without SMOTE, enhance the robustness of the results.

However, the study's single‐center design inherently limits its external validity, despite achieving an AUC exceeding 0.9. Additionally, the reliance on k‐nearest neighbor imputation introduces potential uncertainty in the data, while the use of SMOTE, though effective in addressing class imbalance, may not fully mitigate the impact of the original imbalance on model performance. The complexity of employing multiple ML algorithms further complicates interpretability. Importantly, the absence of external validation restricts the ability to assess the model's applicability to broader populations. Future research should prioritize external validation, particularly, in a multicenter study, and consider alternative imputation techniques to overcome these challenges and enhance generalizability.

A key future direction for this research is the implementation of these ML models into a user‐friendly, automated risk stratification system integrated within electronic health records. Such an integration would enable real‐time identification of patients at higher risk for in‐hospital mortality following AMI, facilitating timely clinical decision‐making and personalized management strategies.

## 5 Conclusion

In conclusion, our study demonstrates the strong performance of ML models in predicting in‐hospital mortality among AMI patients, with the RF model achieving the highest AUC‐ROC (0.900) and specificity (88%). Key predictors such as FBS, LVEF, and serum CR were significant, as highlighted by SHAP analysis, along with age, LDL‐C, and pre‐PCI stenosis. BMI (*p* = 0.456) showed no statistically significant correlation with mortality. The application of SMOTE improved model performance slightly, with RF maintaining an AUC of 0.894 without SMOTE. Logistic regression with LASSO regularization (AUC = 0.865) and SVM with a radial kernel (AUC = 0.875) also performed well, demonstrating the robustness of ML models in handling imbalanced data sets and enhancing risk prediction for AMI.

## Author Contributions

**Hamidreza Soleimani:** conceptualization, methodology, reviewing, and editing the manuscript. **Soroush Najdaghi** and **Delaram Narimani Davani:** writing discussion, abstract, manuscript submission. **Parham Dastjerdi** and **Hamidreza Soleimani:** writing results, supporting materials. **Parham Samimisedeh:** introduction, graphical abstract. **Babak Sattartabar, Farzad Masoudkabir, Haleh Ashraf, Mehdi Mehrani,** and **Yaser Jenab:** data curation, manuscript editing. **Kaveh Hosseini:** conceptualization, supervision, critical revision of the manuscript, final approval.

## Ethics Statement

The co‐authors guarantee the legitimacy and soundness of this study and ensure compliance with all the principles of the Declaration of Helsinki. This study was approved by IRB and the Ethics Committee at THC (Ethical Code: IR.TUMS.THC.REC.1399.045).

## Consent

The authors have nothing to report.

## Conflicts of Interest

The authors declare no conflicts of interest.

## Supporting information


**Supplemental Table 1.** List of variables and their definitions.
**Supplemental Table 2.** Performance comparison of machine learning models for in‐hospital mortality prediction without utilizing SMOTE.
**Supplemental Table 3.** Performance comparison of machine learning models for in‐hospital mortality for the mixed model.
**Supplement Table 4.** Comparison of machine learning model performance across different settings (AUC values).
**Supplemental Table 5.** Performance comparison of machine learning models for in‐hospital mortality prediction utilizing ADASYN (Sensitivity analysis).
**Supplemental Table 6. Performance comparison of machine learning models for in‐hospital mortality prediction utilizing ADASYN (Sensitivity analysis)**.
**Supplement Table 7. Missing data for each variable**.
**Supplement Table 8. Optimal hyperparameters for RF, XGBoost, and SVM**.
Supplemental Figure 1.
Displaying the ROC curves comparing the performance of various models without applying SMOTE (AUC values shown in the figure).
**Supplemental Figure 2.** Displaying the ROC curves comparing the performance of various models in the mixed model (AUC values shown in the figure).
**Supplemental Figure 3.** Precision‐recall curves of various machine learning models for the mixed model (AUC‐PR values are shown in the figure).
**Supplemental Figure 4.** Calibration plot for the main analysis of machine learning models predicting in‐hospital mortality.
**Supplemental Figure 5.** Calibration plot for the mixed model combining GRACE score and key predictors.
**Supplemental Figure 6.** Correlation heatmap representing the relationships between selected variables.
**Supplemental Figure 7.** SHAP Beeswarm Plot for Feature Importance of different variables.
**Supplemental Figure 8.1.** SHAP dependence plot for Age.
**Supplemental Figure 8.2.** SHAP dependence plot for BMI.
**Supplemental Figure 8.3.** SHAP dependence plot for serum creatinine.
**Supplemental Figure 8.4.** SHAP dependence plot for fasting blood glucose.
**Supplemental Figure 8.5.** SHAP dependence plot for LDL‐C.
**Supplemental Figure 8.6.** SHAP dependence plot for LVEF.
**Supplemental Figure 8.7.** SHAP dependence plot for total cholesterol.
**Supplemental Figure 8.8.** SHAP dependence plot for waist circumference.
**Supplemental Figure 9.** Displaying the ROC Curves of Various Machine Learning Models in a subset of patients with STEMI (Sensitivity analysis).
**Supplemental Figure 10.** Feature Importance of Variables Based on the Random Forest Model in a subset of patients with STEMI (Sensitivity analysis).
**Supplemental Figure 11.** Precision‐recall curves of various machine learning models in a subset of patients with STEMI (Sensitivity analysis) (AUC‐PR values shown in the figure).
**Supplemental Figure 12.** Displaying the ROC curves comparing the performance of various models without applying ADASYN (AUC values shown in the figure).
**Supplemental Figure 13.** Cross‐validation results for random forest performance across varying mtry values.
**Supplemental Figure 14.** Cross‐validation results for random forest performance across varying ntree values.

## Data Availability

The data set analyzed in this study is available upon reasonable request from the corresponding author.
