# Supplementary material for: Predicting In‐Hospital Mortality in Patients With Acute Myocardial Infarction: A Comparison of Machine Learning Approaches
Source: Clin Cardiol. 2025 Mar 27;48(4):e70124. doi: 10.1002/clc.70124 (PMC11947610; doi:10.1002/clc.70124)
Supplement: Supplementary file 1 — Supplemental Table 1. List of variables and their definitions. Supplemental Table 2. Performance comparison of machine learning models for in‐hospital mortality prediction without utilizing SMOTE. Supplemental Table 3. Performance comparison of machine learning models for in‐hospital mortality for the mixed model. Supplement Table 4. Comparison of machine learning model performance across different settings (AUC values). Supplemental Table 5. Performance comparison of machine learning models for in‐hospital mortality prediction utilizing ADASYN (Sensitivity analysis). Supplemental Table 6. Performance comparison of machine learning models for in‐hospital mortality prediction utilizing ADASYN (Sensitivity analysis). Supplement Table 7. Missing data for each variable. Supplement Table 8. Optimal hyperparameters for RF, XGBoost, and SVM. Supplemental Figure 1. Displaying the ROC curves comparing the performance of various models without applying SMOTE (AUC values shown in the figure). Supplemental Figure 2. Displaying the ROC curves comparing the performance of various models in the mixed model (AUC values shown in the figure). Supplemental Figure 3. Precision‐recall curves of various machine learning models for the mixed model (AUC‐PR values are shown in the figure). Supplemental Figure 4. Calibration plot for the main analysis of machine learning models predicting in‐hospital mortality. Supplemental Figure 5. Calibration plot for the mixed model combining GRACE score and key predictors. Supplemental Figure 6. Correlation heatmap representing the relationships between selected variables. Supplemental Figure 7. SHAP Beeswarm Plot for Feature Importance of different variables. Supplemental Figure 8.1. SHAP dependence plot for Age. Supplemental Figure 8.2. SHAP dependence plot for BMI. Supplemental Figure 8.3. SHAP dependence plot for serum creatinine. Supplemental Figure 8.4. SHAP dependence plot for fasting blood glucose. Supplemental Figure 8.5. SHAP dependence plot f [file CLC-48-e70124-s001.docx]

**Predicting In-Hospital Mortality After Myocardial Infarction: A Machine Learning Approach**

Supplemental Material

Table of Contents

[Supplemental Table 1. List of variables and their definitions. 3](#_Toc183117890)

[Supplemental Table 2. Performance comparison of machine learning models for in-hospital mortality prediction without utilizing SMOTE 6](#_Toc183117891)

[Supplemental Table 3. Performance comparison of machine learning models for in-hospital mortality for the mixed model 7](#_Toc183117892)

**Supplement Table 4. Comparison of machine learning model performance across different settings (AUC values) ……………………………………………………………………………………………………………………………………………………….. 8**

[Supplemental Table 5. Performance comparison of machine learning models for in-hospital mortality prediction in a subset of patients with STEMI (Sensitivity analysis). 9](#_Toc183117893)

[Supplemental Table 6. Performance comparison of machine learning models for in-hospital mortality prediction utilizing ADASYN (Sensitivity analysis). 10](#_Toc183117894)

**Supplement Table 7. Missing data for each variable …………………………………………………………………..………………11**

**Supplement Table 8. Optimal hyperparameters for RF, XGBoost, and SVM ……………………………………..………..13**

[Statistical Packages 14](#_Toc183117895)

[Supplemental Figure 1. Displaying the ROC curves comparing the performance of various models without applying SMOTE (AUC values shown in the figure). 15](#_Toc183117896)

[Supplemental Figure 2. Displaying the ROC curves comparing the performance of various models in the mixed model (AUC values shown in the figure). 16](#_Toc183117897)

[Supplemental Figure 3. Precision-recall curves of various machine learning models for the mixed model (AUC-PR values are shown in the figure). 17](#_Toc183117898)

[Supplemental Figure 4. Calibration plot for the main analysis of machine learning models predicting in-hospital mortality. 18](#_Toc183117899)

[Supplemental Figure 5. Calibration plot for the mixed model combining GRACE score and key predictors. 19](#_Toc183117900)

[Supplemental Figure 6. Correlation heatmap representing the relationships between selected variables. 20](#_Toc183117901)

[Supplemental Figure 7. SHAP Beeswarm Plot for Feature Importance of different variables. 21](#_Toc183117902)

[SHAP Dependence Plots 22](#_Toc183117903)

[Supplemental Figure 8.1. SHAP dependence plot for Age. 22](#_Toc183117904)

[Supplemental Figure 8.2. SHAP dependence plot for BMI. 23](#_Toc183117905)

[Supplemental Figure 8.3. SHAP dependence plot for serum creatinine. 24](#_Toc183117906)

[Supplemental Figure 8.4. SHAP dependence plot for fasting blood glucose. 25](#_Toc183117907)

[Supplemental Figure 8.5. SHAP dependence plot for LDL-C. 26](#_Toc183117908)

[Supplemental Figure 8.6. SHAP dependence plot for LVEF. 27](#_Toc183117909)

[Supplemental Figure 8.7. SHAP dependence plot for total cholesterol. 28](#_Toc183117910)

[Supplemental Figure 8.8. SHAP dependence plot for waist circumference. 29](#_Toc183117911)

[Sensitivity Analysis 30](#_Toc183117912)

[Supplemental Figure 9. Displaying the ROC Curves of Various Machine Learning Models in a subset of patients with STEMI (Sensitivity analysis). 30](#_Toc183117913)

[Supplemental Figure 10. Feature Importance of Variables Based on the Random Forest Model in a subset of patients with STEMI (Sensitivity analysis). 31](#_Toc183117914)

[Supplemental Figure 11. Precision-recall curves of various machine learning models in a subset of patients with STEMI (Sensitivity analysis) (AUC-PR values shown in the figure). 32](#_Toc183117915)

[Supplemental Figure 12. Displaying the ROC curves comparing the performance of various models without applying ADASYN (AUC values shown in the figure). 33](#_Toc183117916)

[Supplemental Figure 13. Cross-validation results for random forest performance across varying mtry values. 34](#_Toc183117917)

[Supplemental Figure 14. Cross-validation results for random forest performance across varying ntree values. 35](#_Toc183117918)

# Supplemental Table 1. List of variables and their definitions.

| variables |  |
| --- | --- |
| LVEF | Left ventricular ejection fraction (LVEF) is the fraction of chamber volume ejected in systole (stroke volume) in relation to the volume of the blood in the ventricle at the end of diastole (end-diastolic volume) |
| Age | The number of years a person has lived since birth |
| BMI | Body Mass Index, a calculation derived from weight and height( weight (kg)/height² (m²)), utilized to categorize individuals into different weight groups (underweight, normal weight, overweight, or obese) |
| Waist Circumference | A measurement of the abdominal girth at the narrowest part of the waist.WC is used to define central obesity |
| Total Cholesterol | The total concentration of cholesterol in the blood, encompassing LDL, HDL, and VLDL |
| Triglyceride | A triglyceride is an ester derived from glycerol and three fatty acids.Triglycerides are the main constituents of body fat in humans. |
| LDL.C | Low-density lipoprotein is one of the five major groups of lipoprotein that transport all fat molecules around the body in extracellular water. |
| HDL.C | High-density lipoprotein is one of the five major groups of lipoproteins that transport all fat molecules around the body in extracellular water. |
| FBS | Fasting Blood Sugar is a test measuring blood glucose levels after a fasting period. |
| Cr | level of creatinine in the blood |
| Hb | Hemoglobin, a protein in red blood cells that carries oxygen throughout the body. |
| Pre PCI Stenosis | The percentage of arterial narrowing observed in coronary arteries prior to Percutaneous Coronary Intervention |
| Post PCI Stenosis | The residual narrowing in coronary arteries after Percutaneous Coronary Intervention |
| Lesion Length | The length of a coronary atherosclerotic lesion,An area of abnormal or damaged tissue |
| Stent Diameter | The diameter of the coronary stent used during PCI |
| Contrast Volume | The volume of contrast agent used during PCI |
| History of STEMI | Previous occurrence of ST-Elevation Myocardial Infarction |
| History of NSTEMI | Previous occurrence of Non-ST Elevation Myocardial Infarction |
| History of UA | History of Unstable Angina |
| History of CCS | History of Chronic Coronary Syndrome |
| Sex | Biological gender |
| Family History of CAD | Family history of Coronary Artery Disease |
| Dyslipidemia | A disorder characterized by abnormal lipid levels in the bloodstream |
| DM | Diabetes mellitus (DM), a chronic metabolic condition, is a disease of inadequate control of blood levels of glucose. |
| Hypertension | Persistently high systemic arterial BLOOD PRESSURE. hypertension is currently defined as when SYSTOLIC PRESSURE is consistently greater than 140 mm Hg or when DIASTOLIC PRESSURE is consistently 90 mm Hg or more. |
| Smoking | Tobacco use |
| Opium | The use of opium |
| History of HFrEF | History of Heart Failure with Reduced Ejection Fraction |
| History of VHD | History of Valvular Heart Disease |
| History of CVA/TIA | History of Cerebrovascular Accident (Stroke) or Transient Ischemic Attack |
| History of Chronic Pulmonary Disease | History of a long-term respiratory condition affecting lung function, such as COPD |
| History of PAD | History of Peripheral Artery Disease |
| History of ESRD | History of End-Stage Renal Disease |
| History of Dialysis | history of requiring dialysis treatment |
| History of CPR | History of Cardiopulmonary Resuscitation |
| Previous CABG | Previous Coronary Artery Bypass Grafting |
| Previous PCI | Previous Percutaneous Coronary Intervention |
| Atrial Fibrillation | Abnormal cardiac rhythm that is characterized by rapid, uncoordinated firing of electrical impulses in the upper chambers of the heart (HEART ATRIA). |
| Lesion Type (ACC/AHA/…) | Classification of coronary lesions by the American College of Cardiology/American Heart Association |
| Pre PCI TIMI Flow | Thrombolysis In Myocardial Infarction flow grade prior to PCI |
| Post PCI TIMI Flow | Thrombolysis In Myocardial Infarction flow grade after PCI |
| Complications (Coronary Perforation) | A rare but serious complication of PCI where the coronary artery is perforated, leading to potential life-threatening consequences |
| Complications (Coronary Dissection) | The tearing of the coronary artery wall during PCI, which can lead to compromised blood flow |
| Complications (No reflow/Slow reflow) | A phenomenon where blood flow is not adequately restored after PCI |
| Complications (Side Branch Occlusion) | The inadvertent occlusion of a coronary side branch during PCI |
| Complications (Stroke) | An ischemic or hemorrhagic stroke occurring as a complication of PCI |
| Shock | Shock is characterized by decreased oxygen delivery and/or increased oxygen consumption or inadequate oxygen utilization leading to cellular and tissue hypoxia. |
| Access Site Complications | Complications arising from the arterial or venous access site used during PCI |
| Pseudo Aneurysm | a leakage of arterial blood from an artery into the surrounding tissue with a persistent communication between the originating artery and the resultant adjacent cavity |
| Hematoma | A localized collection of blood at the access site |
| Major Bleeding | Significant hemorrhage, often requiring transfusion or surgical intervention. |
| Need for Transfusion | Requirement of blood transfusion due to excessive blood loss during or after PCI |
| Elevated Biomarkers | An increase in cardiac biomarkers post-PCI |
| Post PCI STEMI | Occurrence of ST-Elevation Myocardial Infarction after PCI |
| Post PCI AKI Requiring Dialysis | Acute Kidney Injury following PCI severe enough to necessitate dialysis |
| Post PCI Tamponade | Occurrence of Tamponade, fluid accumulation in the pericardial sac, after PCI |
| CAD Extension | Progression of Coronary Artery Disease beyond the originally treated area |
| Left Main Coronary Artery Lesion | A blockage or narrowing of the left main coronary artery |

##

# Supplemental Table 2. Performance comparison of machine learning models for in-hospital mortality prediction without utilizing SMOTE

| Model Name | AUC (95% CI) | Sensitivity | Specificity | Brier Score | F1 Score |
| --- | --- | --- | --- | --- | --- |
| Logistic Regression with Forward Selection | 0.893(0.842-0.943) | 0.81 | 0.85 | 0.014 | 0.992 |
| Logistic Regression with Lasso Selection | 0.9(0.859-0.942) | 0.83 | 0.79 | 0.014 | 0.992 |
| Neural Network | 0.905(0.869-0.941) | 0.92 | 0.73 | 0.014 | 0.992 |
| Random Forest | 0.905(0.865-0.944) | 0.86 | 0.8 | 0.014 | 0.992 |
| Extreme Gradient Boosting | 0.884(0.835-0.933) | 0.83 | 0.81 | 0.015 | 0.992 |
| SVM - Linear Kernel | 0.732(0.651-0.813) | 0.69 | 0.68 | 0.016 | 0.992 |
| SVM - Radial Kernel | 0.848(0.773-0.922) | 0.86 | 0.75 | 0.015 | 0.991 |

# Supplemental Table 3. Performance comparison of machine learning models for in-hospital mortality for the mixed model

| Model Name | AUC (95% CI) | Sensitivity | Specificity | Brier Score | F1 Score |
| --- | --- | --- | --- | --- | --- |
| Logistic Regression with Forward Selection | 0.872(0.812-0.933) | 0.92 | 0.7 | 0.015 | 0.992 |
| Logistic Regression with Lasso Selection | 0.871(0.81-0.932) | 0.92 | 0.7 | 0.015 | 0.991 |
| Neural Network | 0.8(0.724-0.876) | 0.69 | 0.8 | 0.018 | 0.99 |
| Random Forest | 0.853(0.788-0.917) | 0.86 | 0.69 | 0.015 | 0.992 |
| Extreme Gradient Boosting | 0.834(0.762-0.906) | 0.72 | 0.82 | 0.015 | 0.991 |
| SVM - Linear Kernel | 0.721(0.617-0.826) | 0.75 | 0.71 | 0.016 | 0.992 |
| SVM - Radial Kernel | 0.711(0.62-0.802) | 0.61 | 0.75 | 0.017 | 0.99 |

**Supplement Table 4.** Comparison of machine learning model performance across different settings (AUC values)

| Setting | RF | XGBoost | LR-Lasso | LR-Forward | NN | SVM-R | SVM-L |
| --- | --- | --- | --- | --- | --- | --- | --- |
| Mixed | 0.853 | 0.834 | 0.871 | 0.872 | 0.8 | 0.711 | 0.721 |
| Main | 0.924 | 0.905 | 0.893 | 0.882 | 0.878 | 0.862 | 0.866 |
| Without SMOTE | 0.905 | 0.884 | 0.9 | 0.893 | 0.905 | 0.848 | 0.732 |
| Average | 0.894 | 0.874 | 0.888 | 0.882 | 0.861 | 0.807 | 0.773 |

**Abbreviations: AUC**: Area Under the Curve; **LR-Lasso**: Logistic Regression with Least Absolute Shrinkage and Selection Operator; **LR-Forward**: Logistic Regression with Forward Selection; **NN**: Neural Network; **RF**: Random Forest; **SMOTE**: Synthetic Minority Oversampling Technique; **SVM-L**: Support Vector Machine with Linear Kernel; **SVM-R**: Support Vector Machine with Radial Kernel; **XGBoost**: Extreme Gradient Boosting

#

# Supplemental Table 4. Performance comparison of machine learning models for in-hospital mortality prediction in a subset of patients with STEMI (Sensitivity analysis).

| Model Name | AUC (95% CI) | Sensitivity | Specificity | Brier Score | F1 Score |
| --- | --- | --- | --- | --- | --- |
| Logistic Regression with Forward Selection | 0.882(0.826-0.938) | 0.78 | 0.87 | 0.022 | 0.987 |
| Logistic Regression with Lasso Selection | 0.891(0.942-0.939) | 0.81 | 0.82 | 0.021 | 0.988 |
| Neural Network | 0.854(0.800-0.909) | 0.81 | 0.74 | 0.026 | 0.984 |
| Random Forest | 0.9(0.855-0.944) | 0.83 | 0.85 | 0.021 | 0.987 |
| Extreme Gradient Boosting | 0.901(0.860-0.943) | 0.89 | 0.79 | 0.021 | 0.987 |
| SVM - Linear Kernel | 0.67(0.567-0.774) | 0.5 | 0.82 | 0.025 | 0.987 |
| SVM - Radial Kernel | 0.83(0.763-0.896) | 0.83 | 0.75 | 0.026 | 0.984 |

# Supplemental Table 5. Performance comparison of machine learning models for in-hospital mortality prediction utilizing ADASYN (Sensitivity analysis).

| Model Name | AUC (95% CI) | Sensitivity | Specificity | Brier Score | F1 Score |
| --- | --- | --- | --- | --- | --- |
| Logistic Regression with Forward Selection | 0.839(0.771-0.907) | 0.79 | 0.8 | 0.019 | 0.99 |
| Logistic Regression with Lasso Selection | 0.844(0.777-0.910) | 0.74 | 0.85 | 0.018 | 0.991 |
| Neural Network | 0.822(0.758-0.886) | 0.82 | 0.73 | 0.022 | 0.986 |
| Random Forest | 0.887(0.840-0.933) | 0.85 | 0.81 | 0.016 | 0.991 |
| Extreme Gradient Boosting | 0.852(0.793-0.911) | 0.79 | 0.79 | 0.017 | 0.991 |
| SVM - Linear Kernel | 0.841(0.780-0.929) | 0.74 | 0.83 | 0.017 | 0.991 |
| SVM - Radial Kernel | 0.752(0.658-0.844) | 0.74 | 0.75 | 0.019 | 0.989 |

**Supplement Table 6. Missing data for each variable**

| **Variables** | **Missing_Percentage** |
| --- | --- |
| LVEF | 0 |
| Age | 0 |
| BMI | 0 |
| Waist.Circumference | 0 |
| Total.Cholesterol | 0 |
| Triglyceride | 0 |
| LDL.C | 0 |
| HDL.C | 0 |
| FBS | 0.021926619 |
| Cr | 0.007308873 |
| Hb | 0.109633095 |
| Pre.PCI.Stenosis | 0.021926619 |
| Post.PCI.Stenosis | 0.102324222 |
| Lesion.Length | 1.439847975 |
| Stent.Diameter | 7.805876334 |
| Contrast.Volume | 9.457681625 |
| Historyf.STEMI | 8.470983774 |
| History.of.NSTEMI | 0 |
| History.of.UA | 0.116941968 |
| History.of.CCS | 0.182721824 |
| Sex | 0 |
| Family.History.of.CAD | 0 |
| Dyslipidemia | 0 |
| DM | 0 |
| Hypertension | 0 |
| Smoking | 0 |
| Opium | 0 |
| History.of.HFrEF | 0 |
| History.of.VHD | 0 |
| History.of.CVA.TIA | 0 |
| History.of.Chronic.Pulmonary.Disease | 0 |
| History.of.PAD | 0 |
| History.of.ESRD | 0 |
| History.of.Dialysis | 0 |
| History.of.CPR | 0 |
| Previous.CABG | 0 |
| Previous.PCI | 0 |
| Atrial.Fibrillation | 0 |
| Lesion.Type..ACC.AHA. | 0.328899284 |
| Pre.PCI.TIMI.Flow | 0.328899284 |
| Post.PCI.TIMI.Flow | 0.328899284 |
| Complications...Coronary.Perforation | 0.657798567 |
| Complications..Coronary.Dissection | 0.789358281 |
| Complications...No.reflow.Slow.reflow | 0.716269551 |
| Complications...Side.Branch.Occlusion | 0.906300249 |
| Complications...Stroke | 0.898991376 |
| Shock | 0.920917994 |
| Access.Site.Complications | 0 |
| Pseudo.Aneurysm | 0 |
| Hematoma | 0 |
| Major.Bleeding | 0 |
| Need.for.Transfusion | 0 |
| Elevated.Biomarkers | 0 |
| Post.PCI.STEMI | 0 |
| Post.PCI.AKI.Requiring.Dialysis | 0 |
| Post.PCI.Tamponade | 0 |
| CAD.Extension | 0 |
| Left.Main.Coronary.Artery.Lesion | 0 |

**Supplement Table 7. Optimal hyperparameters for RF, XGBoost, and SVM**

| **Model** | **Parameter** | **Optimal Value** |
| --- | --- | --- |
| Random Forest | Number of Decision Trees | 6000 |
|  | Maximum Features | 3 |
| Extreme Gradient Boosting (XGBoost) | Number of Boosting Rounds | 1000 |
|  | Alpha | 0.0876 |
|  | Learning Rate | 0.015 |
|  | Lambda | 0.00457 |
| SVM (Linear) | Cost | 0.56961 |
| SVM (Radial) | Cost | 0.1032 |
|  | Sigma | 0.01624 |
| LASSO | Alpha | 0.06715 |
|  | Lambda | 0.04755 |
| Neural Network | Size | 3 |
|  | Decay | 0.0124 |

# Statistical Packages

| Pacakge Name | Application | URL |
| --- | --- | --- |
| tidyverse | The tidyverse is an opinionated collection of R packages designed for data science. | <https://cran.r-project.org/web/packages/tidyverse/index.html> |
| caret | The caret package (short for Classification And REgression Training) is a set of functions that attempt to streamline the process for creating predictive models. | <https://cran.r-project.org/web/packages/caret/index.html> |
| rsample | The rsample package provides functions to create different types of resamples and corresponding classes for their analysis. | <https://cran.r-project.org/web/packages/rsample/index.html> |
| e1071 | Set of function for implementation of support vector machine algorithms. | <https://cran.r-project.org/web/packages/e1071/index.html> |
| randomForest | Implements Breiman's random forest algorithm (based on Breiman and Cutler's original Fortran code) for classification and regression. | <https://cran.r-project.org/web/packages/randomForest/index.html> |
| xgboost | Package for implementation of the gradient boosting framework | <https://cran.r-project.org/web/packages/xgboost/index.html> |
| nnet | Pacakge for feed-forward neural networks with a single hidden layer, and for multinomial log-linear models. | <https://cran.r-project.org/web/packages/nnet/index.html> |
| glment | Procedures for fitting the entire lasso or elastic-net regularization path for linear regression, logistic and multinomial regression models | <https://cran.r-project.org/web/packages/glmnet/index.html> |
| pROC | Tools for visualizing, smoothing and comparing receiver operating characteristic (ROC curves). | <https://cran.r-project.org/web/packages/pROC/index.html> |
| Mleval | Package for evaluation of machine learning models. | <https://cran.r-project.org/web/packages/MLeval/index.html> |
| themis | Package for implemeting SMOTE resampling methods in unbalanced datasets | <https://cran.r-project.org/web/packages/themis/index.html> |

#
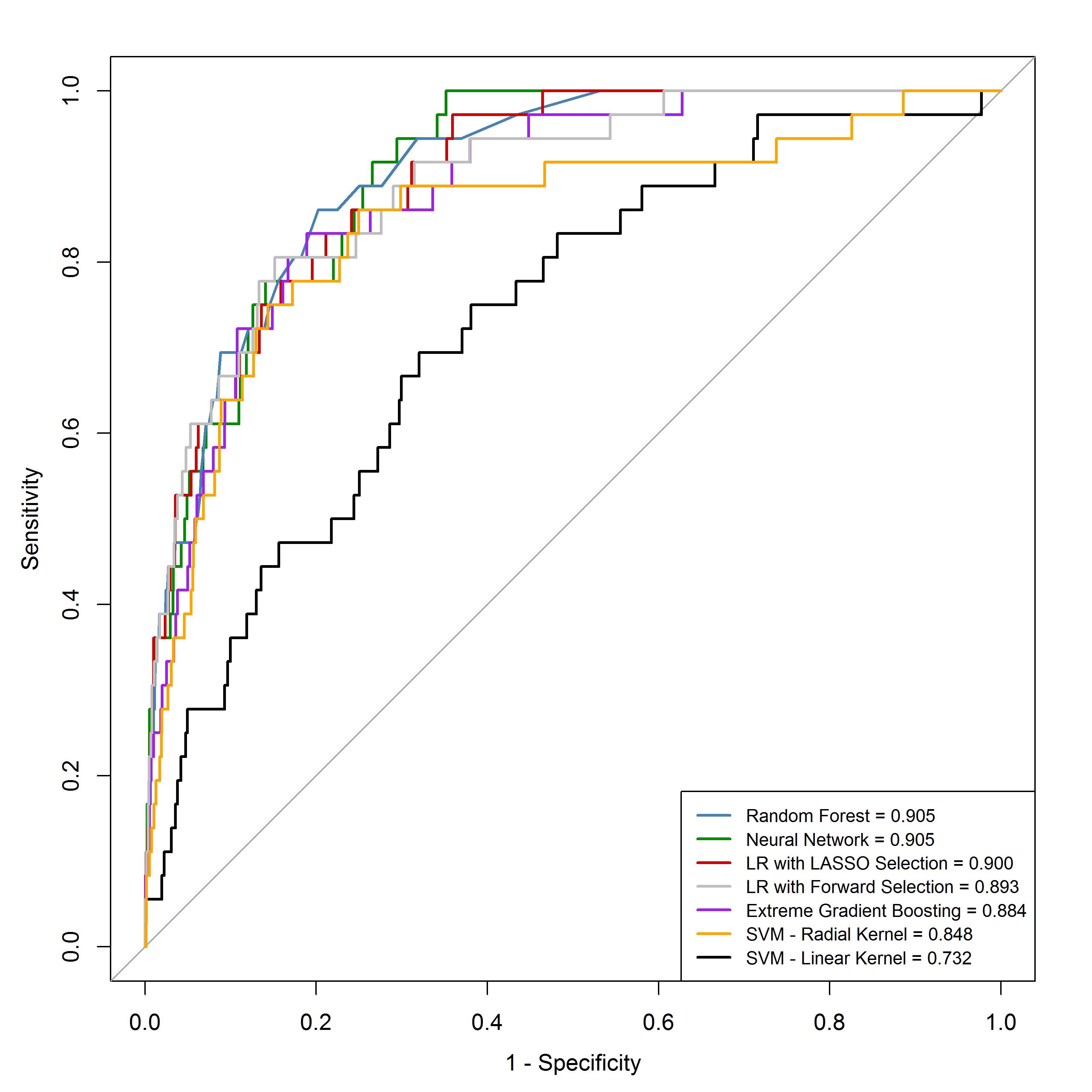
Supplemental Figure 1. Displaying the ROC curves comparing the performance of various models without applying SMOTE (AUC values shown in the figure).

This figure shows the AUC-ROC curves for various models predicting in-hospital mortality in AMI patients without applying SMOTE. The random forest model and the neural network achieved the highest AUC (0.905 each), followed by logistic regression with Lasso selection (AUC = 0.900), logistic regression with forward selection (AUC = 0.893), and extreme gradient boosting (AUC = 0.884). SVM with a radial kernel achieved an AUC of 0.848, while SVM with a linear kernel demonstrated the lowest AUC of 0.732.

# Supplemental Figure 2. Displaying the ROC curves comparing the performance of various models in the mixed model (AUC values shown in the figure).

This figure shows the AUC-ROC curves for various models predicting in-hospital mortality in AMI patients using the mixed model. Logistic regression with forward selection achieved an AUC of 0.872, closely followed by logistic regression with Lasso selection (AUC = 0.871). Random Forest had an AUC of 0.853, and extreme gradient boosting recorded an AUC of 0.834. The neural network model achieved an AUC of 0.800, while SVM with linear and radial kernels demonstrated the lowest performance, with AUCs of 0.721 and 0.711, respectively.

**
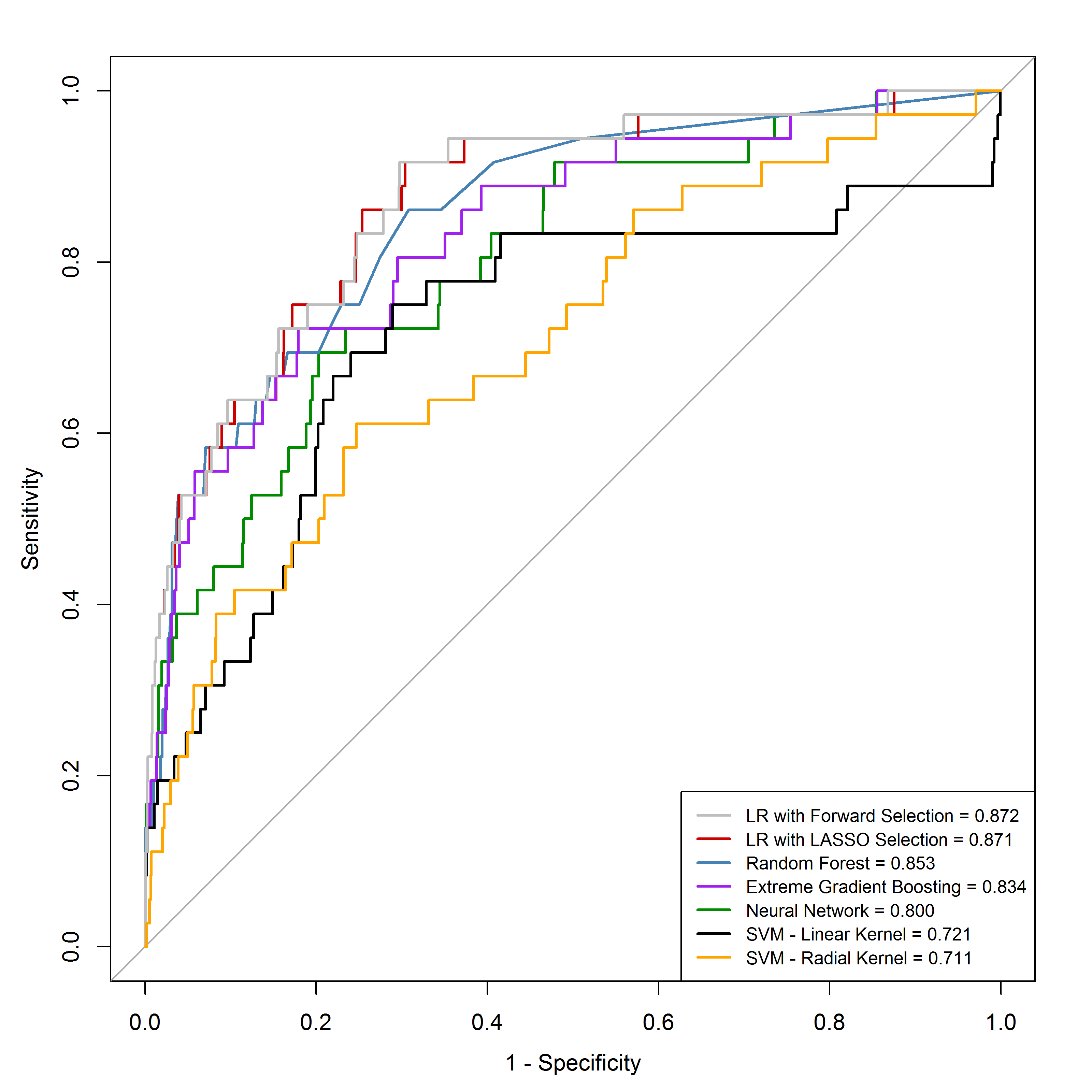
**

#
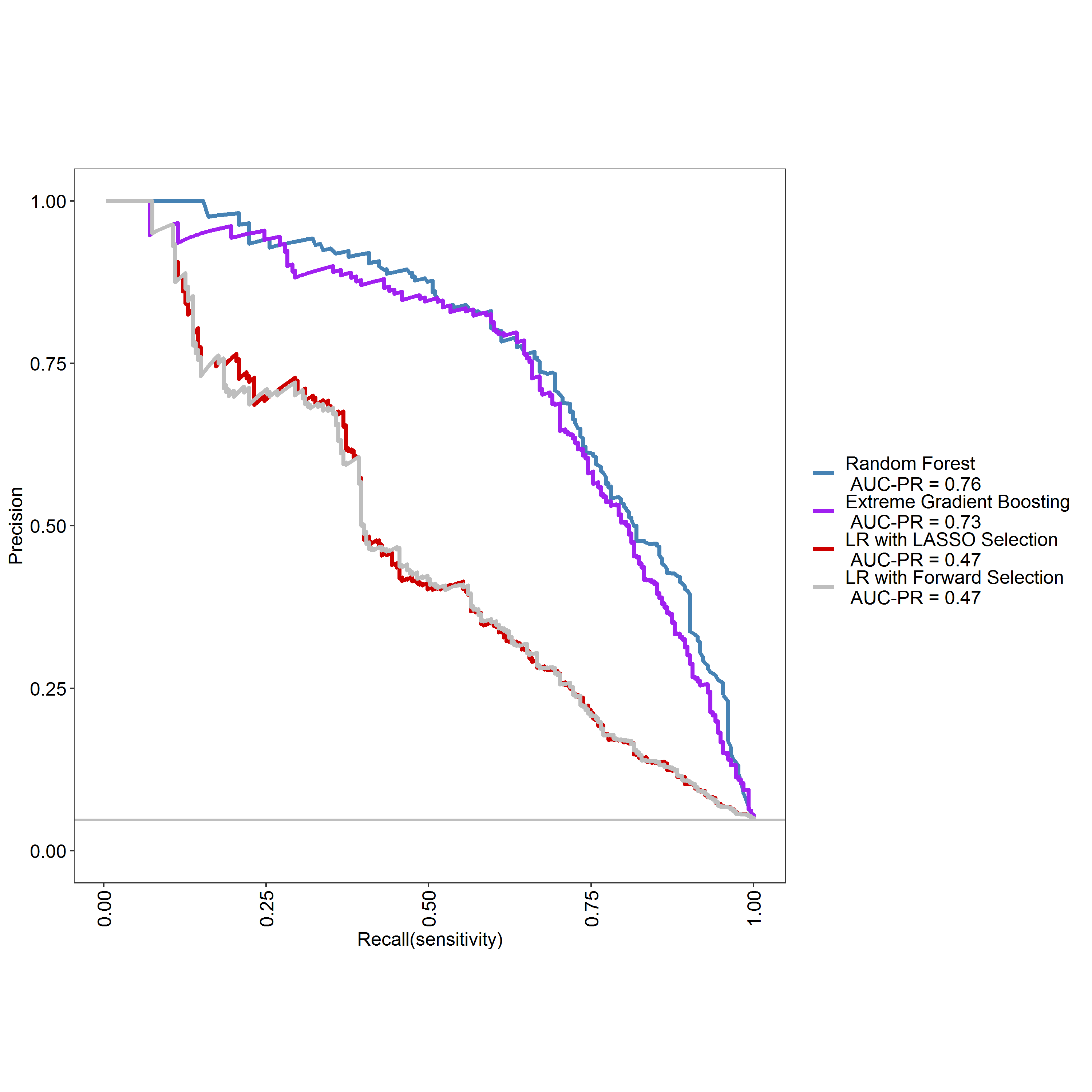
Supplemental Figure 3. Precision-recall curves of various machine learning models for the mixed model (AUC-PR values are shown in the figure).

This figure displays the precision-recall (PR) curves for several machine learning models predicting in-hospital mortality among AMI patients using the mixed model. The random forest model achieved the highest precision-recall area under the curve (AUC-PR = 0.76), followed by extreme gradient boosting (AUC-PR = 0.73). Logistic regression (LR) with Lasso selection and LR with forward selection both recorded an AUC-PR of 0.47.

# Supplemental Figure 4. Calibration plot for the main analysis of machine learning models predicting in-hospital mortality.

**
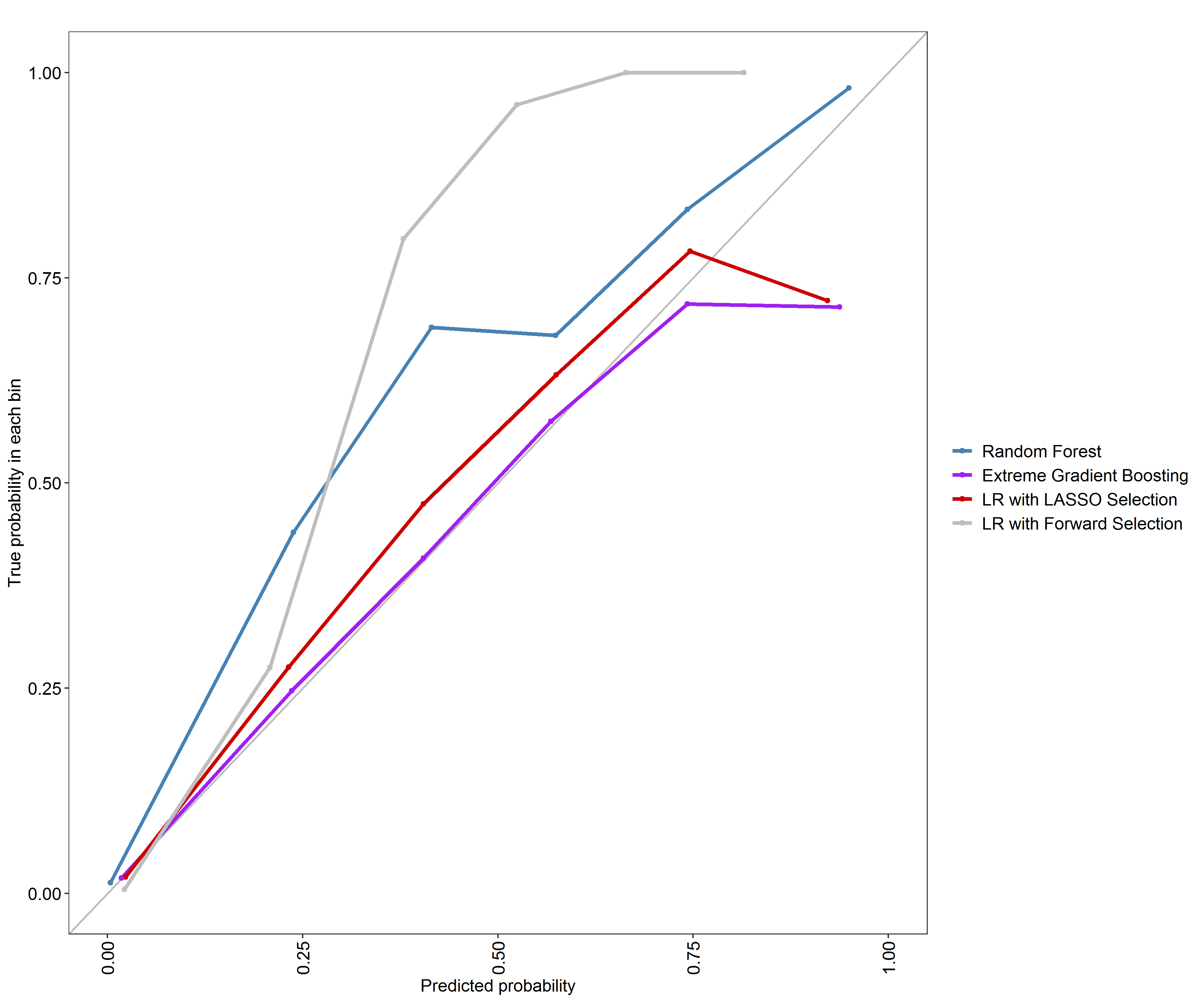
**

# Supplemental Figure 5. Calibration plot for the mixed model combining GRACE score and key predictors.


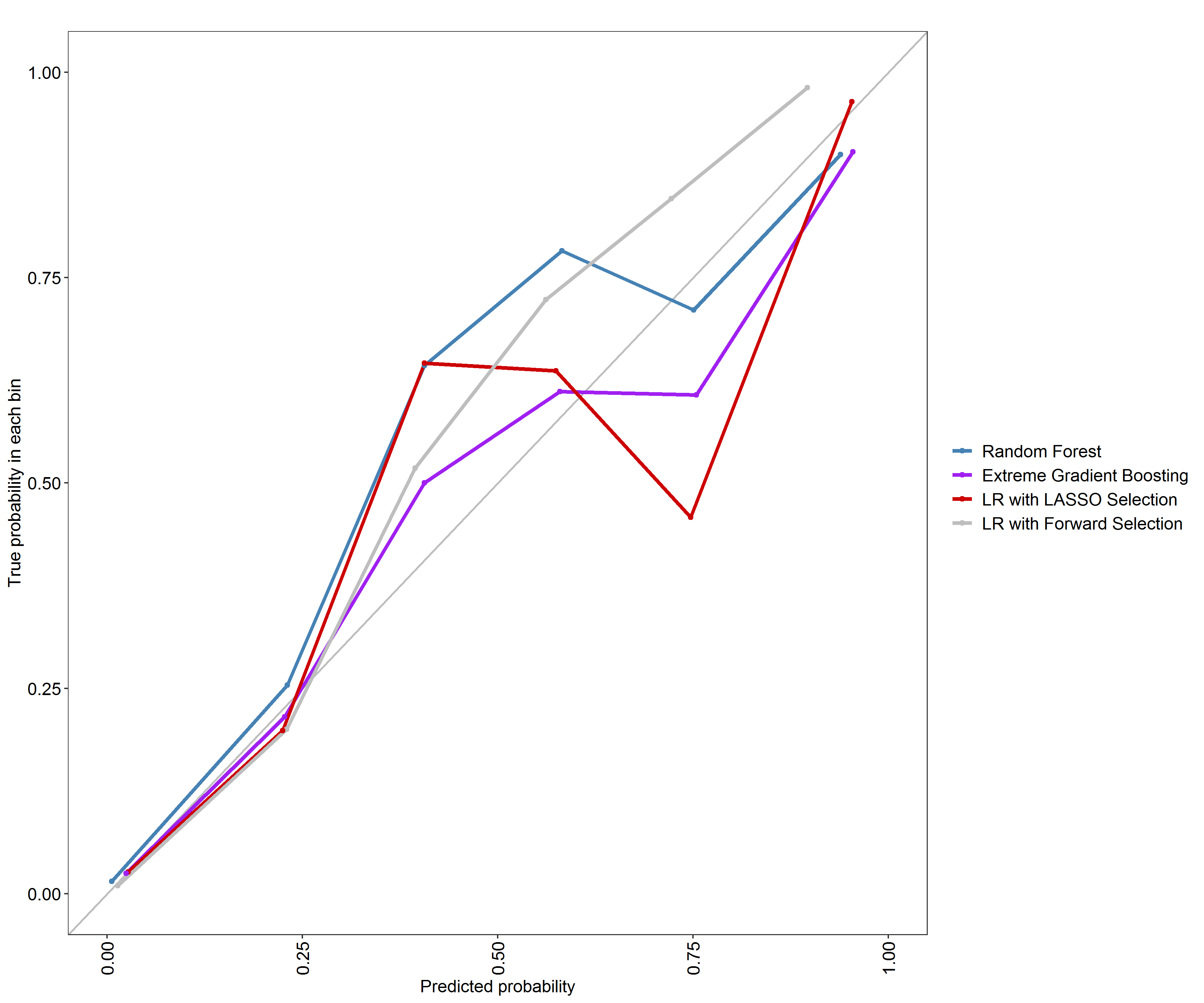


# Supplemental Figure 6. Correlation heatmap representing the relationships between selected variables.

This heatmap displays the correlations between key clinical and biochemical variables in AMI patients. Strong positive correlations are observed between LDL-C and total cholesterol, BMI and waist circumference, and other related variables. The color scale ranges from -1 (negative correlation, red) to 1 (positive correlation, blue).


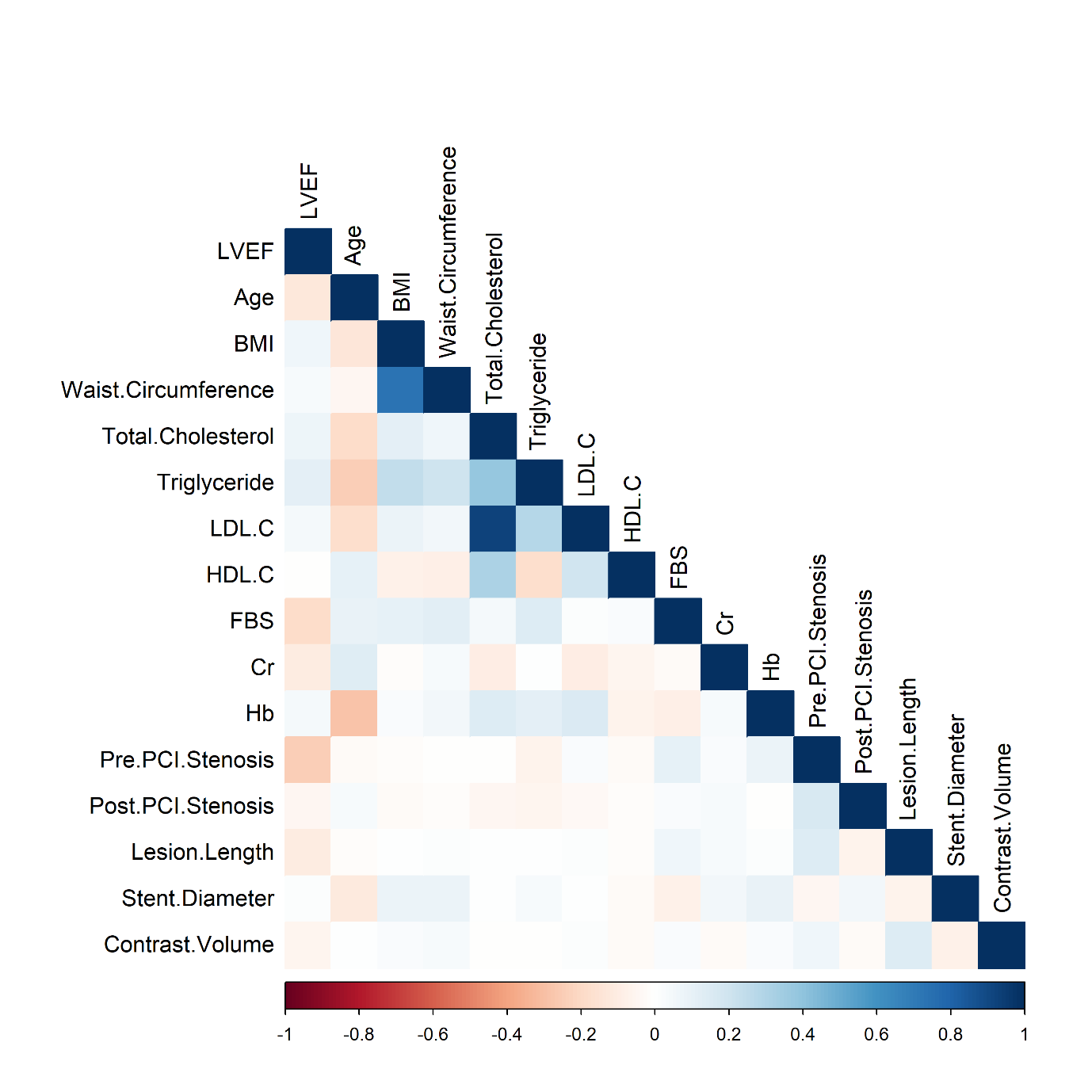


# Supplemental Figure 7. SHAP Beeswarm Plot for Feature Importance of different variables.


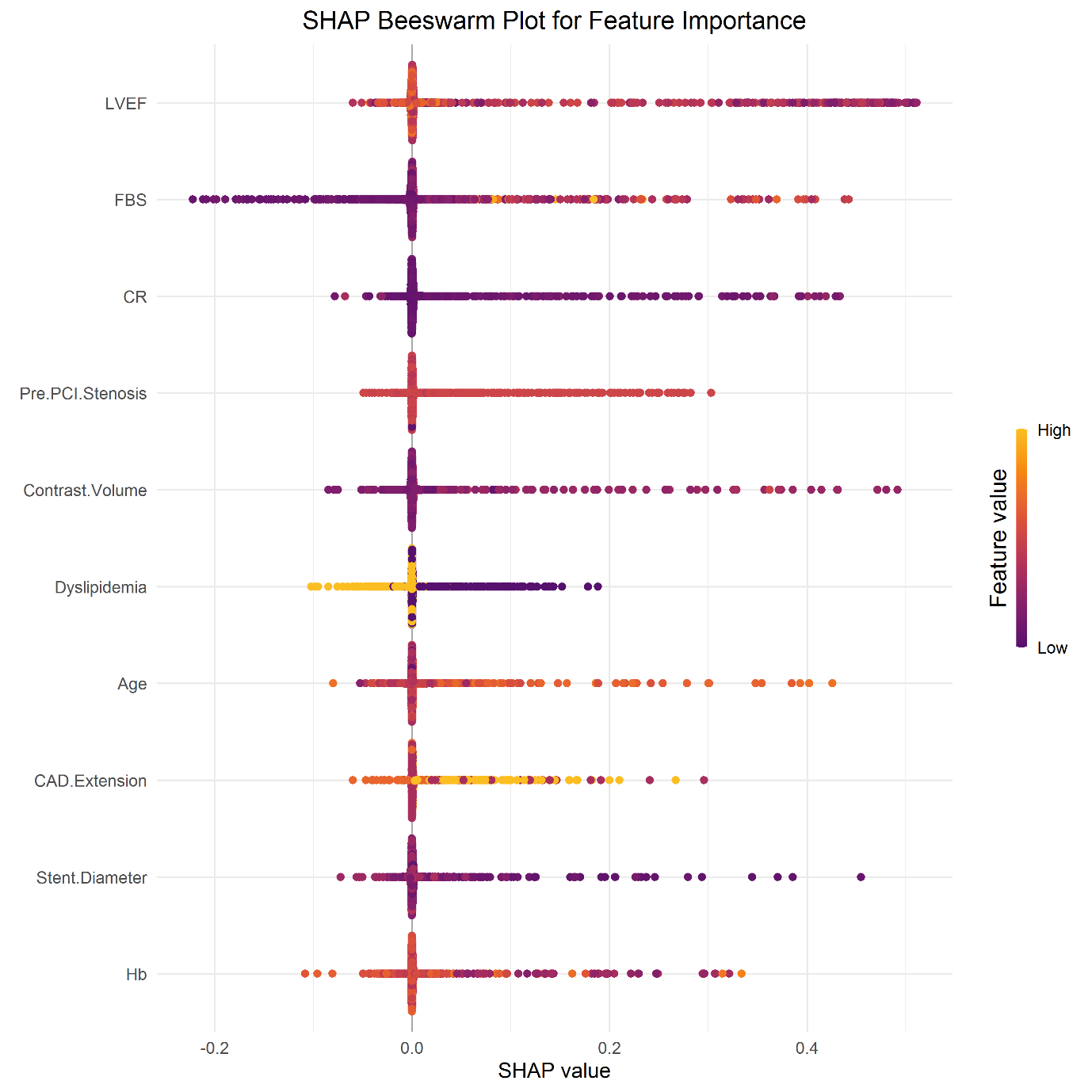


This SHAP (SHapley Additive exPlanations) beeswarm plot illustrates the most influential predictors contributing to the XGBoost model's predictions of in-hospital mortality among AMI patients. Each dot represents an individual patient, with the color gradient indicating feature values (yellow for higher values and purple for lower values). The horizontal position of each dot corresponds to the SHAP value, showing the impact of the feature on the model's prediction. Key predictors include left ventricular ejection fraction (LVEF), fasting blood sugar (FBS), serum creatinine (Cr), pre-PCI stenosis, and contrast volume, among others. Higher SHAP values (positioned to the right) indicate an increased mortality risk, while lower SHAP values (positioned to the left) correspond to a decreased risk. This plot provides a visual summary of how individual feature values influence the model’s predictions.

# **SHAP Dependence Plots**

___________________________________________________________________________

Supplemental Figure 8.1. SHAP dependence plot for Age.


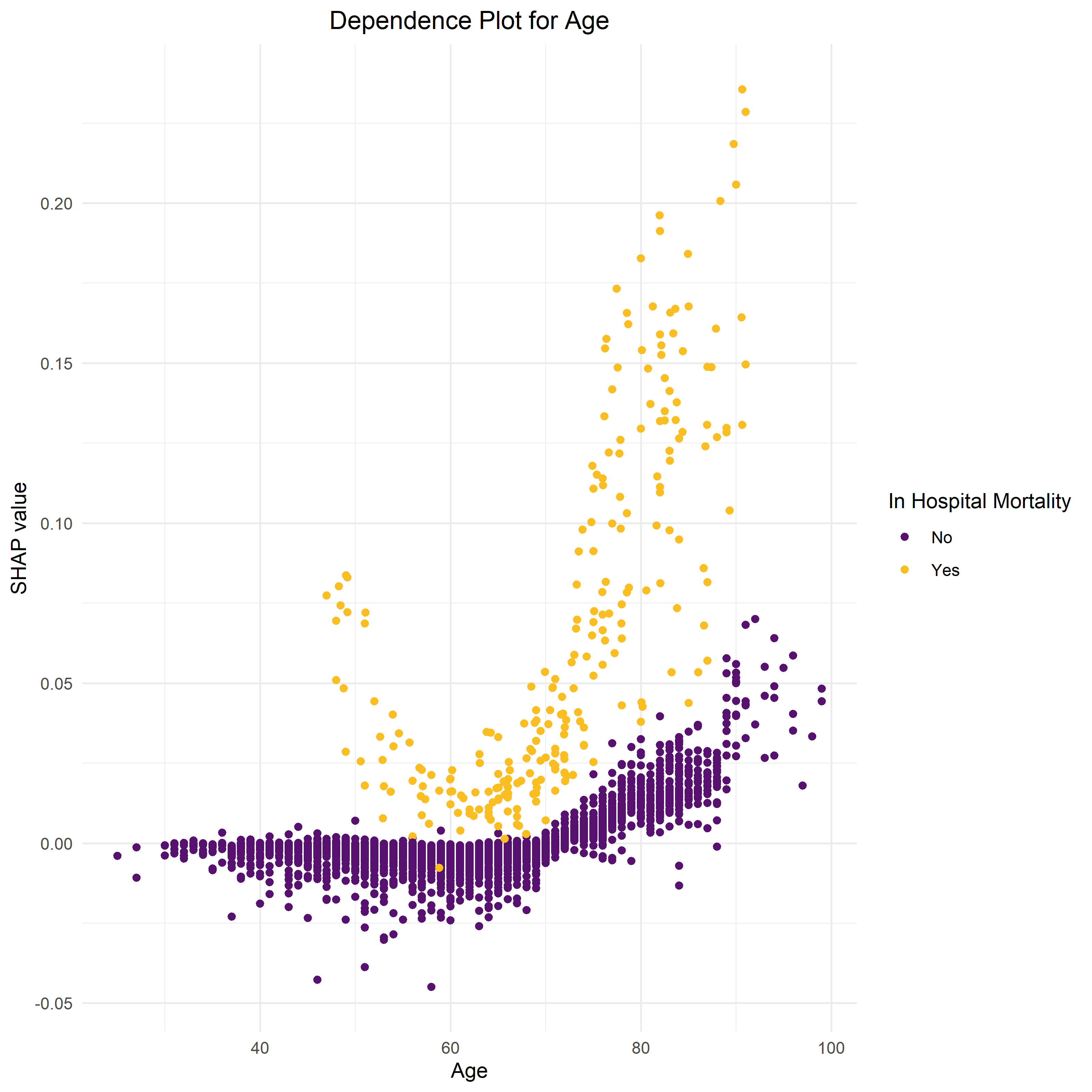


This plot shows the relationship between age and SHAP values in predicting in-hospital mortality in AMI patients. Higher SHAP values correspond to an increased risk of mortality. Yellow dots indicate patients who died in the hospital, while purple dots represent survivors. The U-shaped pattern suggests that both very young and older age groups contribute differently to mortality risk.

## **Supplemental Figure 8.2.** SHAP dependence plot for BMI.

This plot depicts the relationship between BMI and SHAP values in predicting in-hospital mortality for AMI patients. The SHAP values increase slightly as BMI rises, indicating that higher BMI may be associated with a modestly increased mortality risk. Yellow dots represent patients who died in the hospital, while purple dots represent survivors.


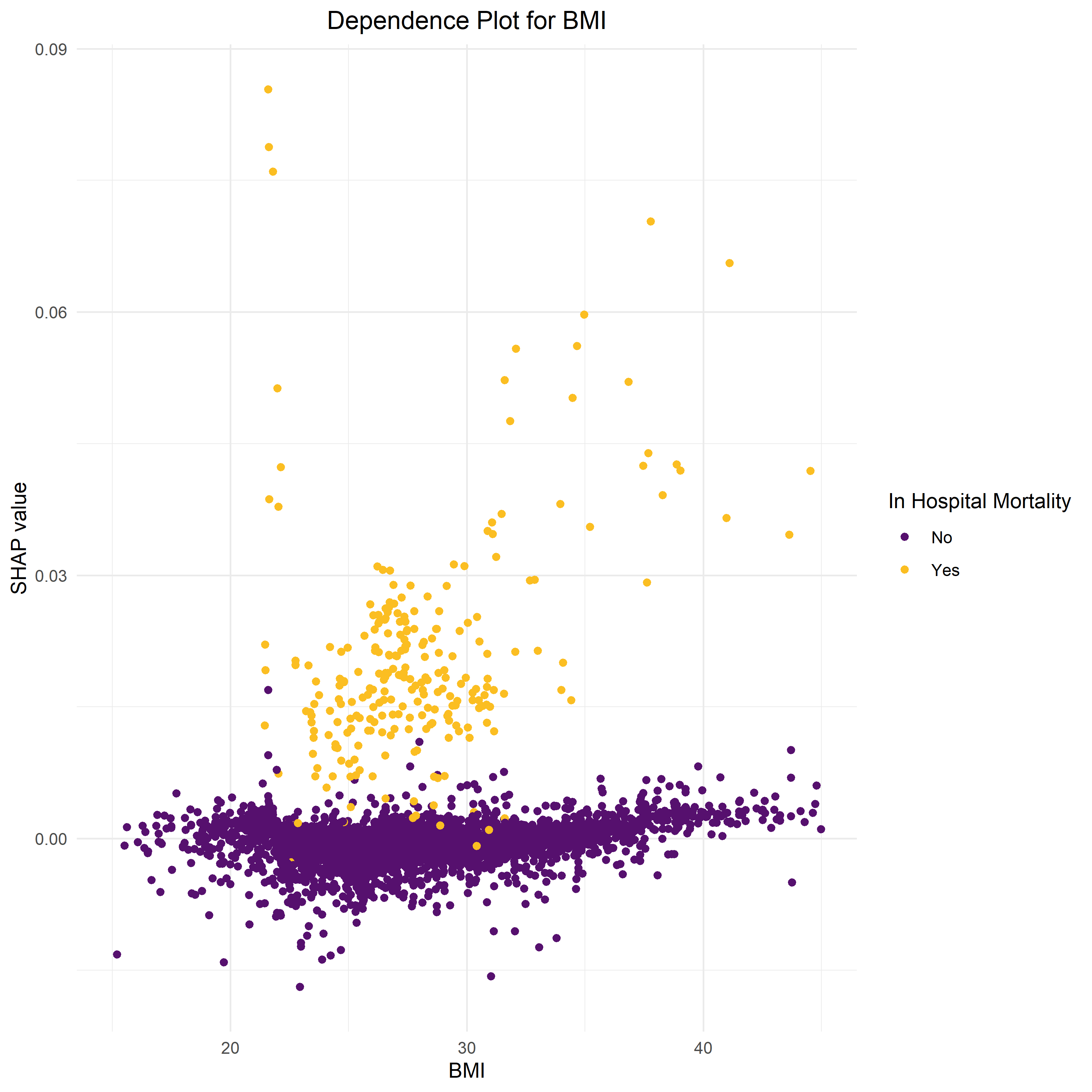


## **Supplemental Figure 8.3.** SHAP dependence plot for serum creatinine.

This plot illustrates the effect of serum creatinine levels on in-hospital mortality prediction in AMI patients. As serum creatinine increases, SHAP values rise, indicating a higher mortality risk. Yellow dots represent patients who died in the hospital, while purple dots represent survivors.


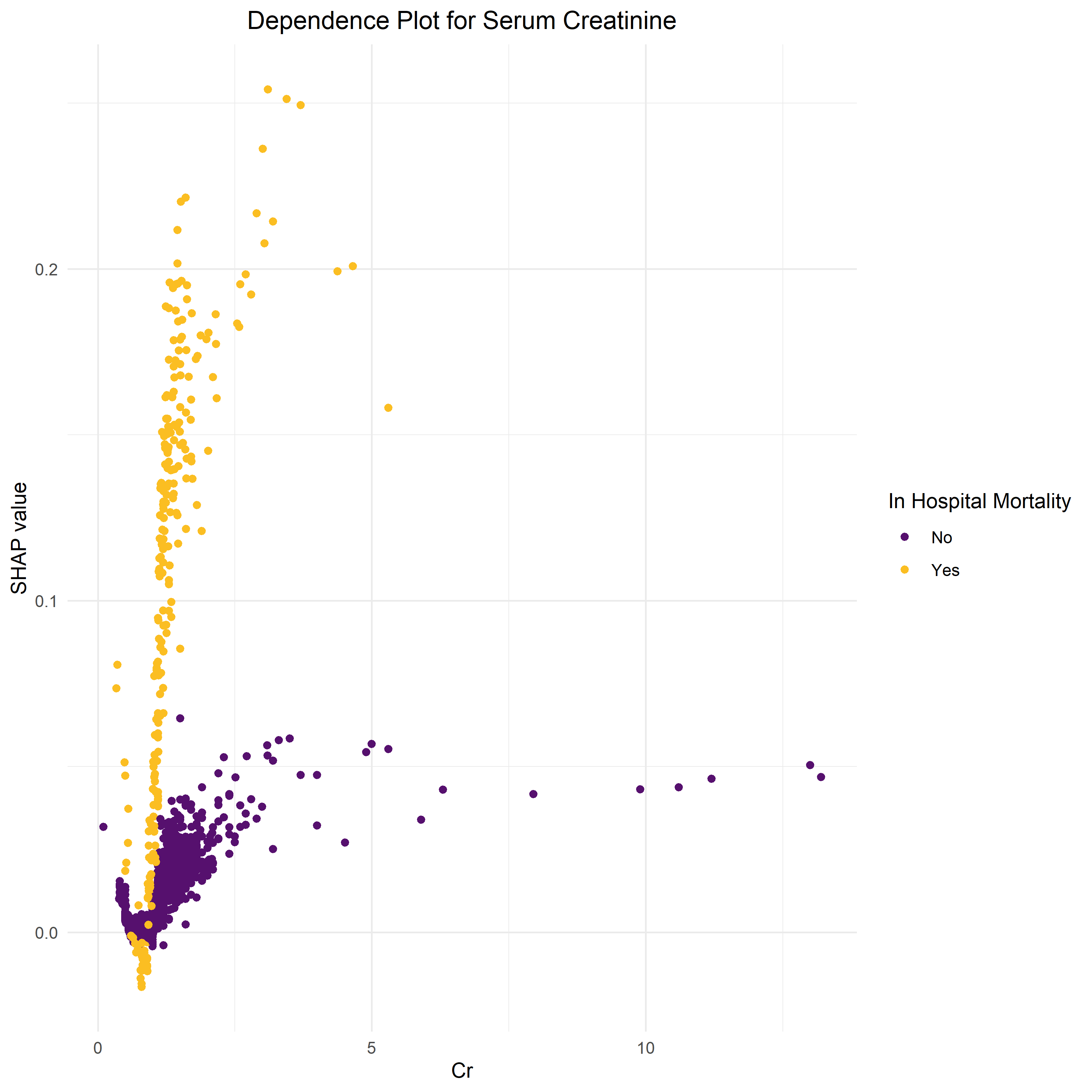


## **Supplemental Figure 8.4.** SHAP dependence plot for fasting blood glucose.

This plot shows the relationship between fasting blood glucose levels and SHAP values in predicting in-hospital mortality for AMI patients. Higher FBS values are associated with increased SHAP values, indicating a higher risk of mortality. Yellow dots represent patients who died in the hospital, while purple dots represent survivors.


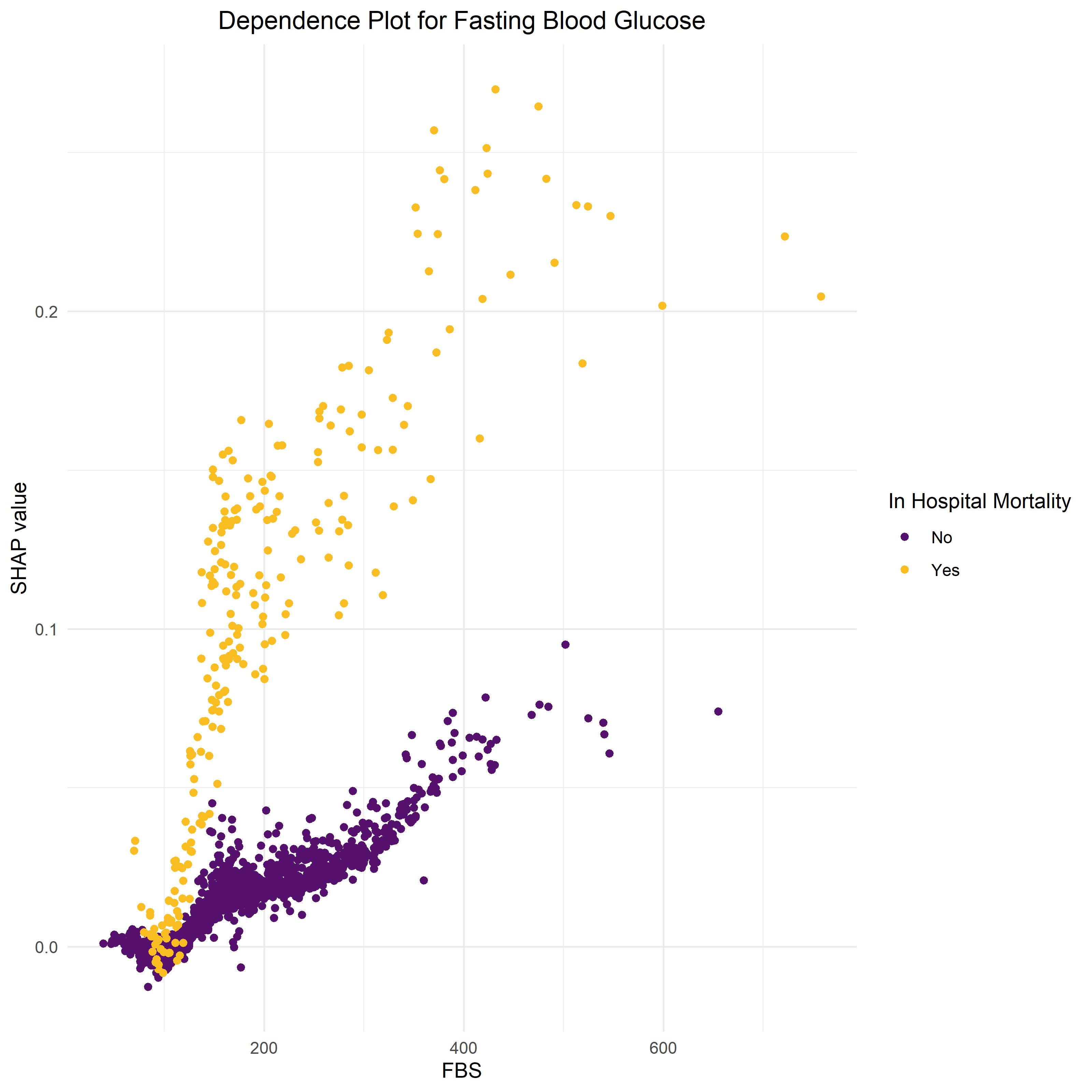


## **Supplemental Figure 8.5.** SHAP dependence plot for LDL-C.


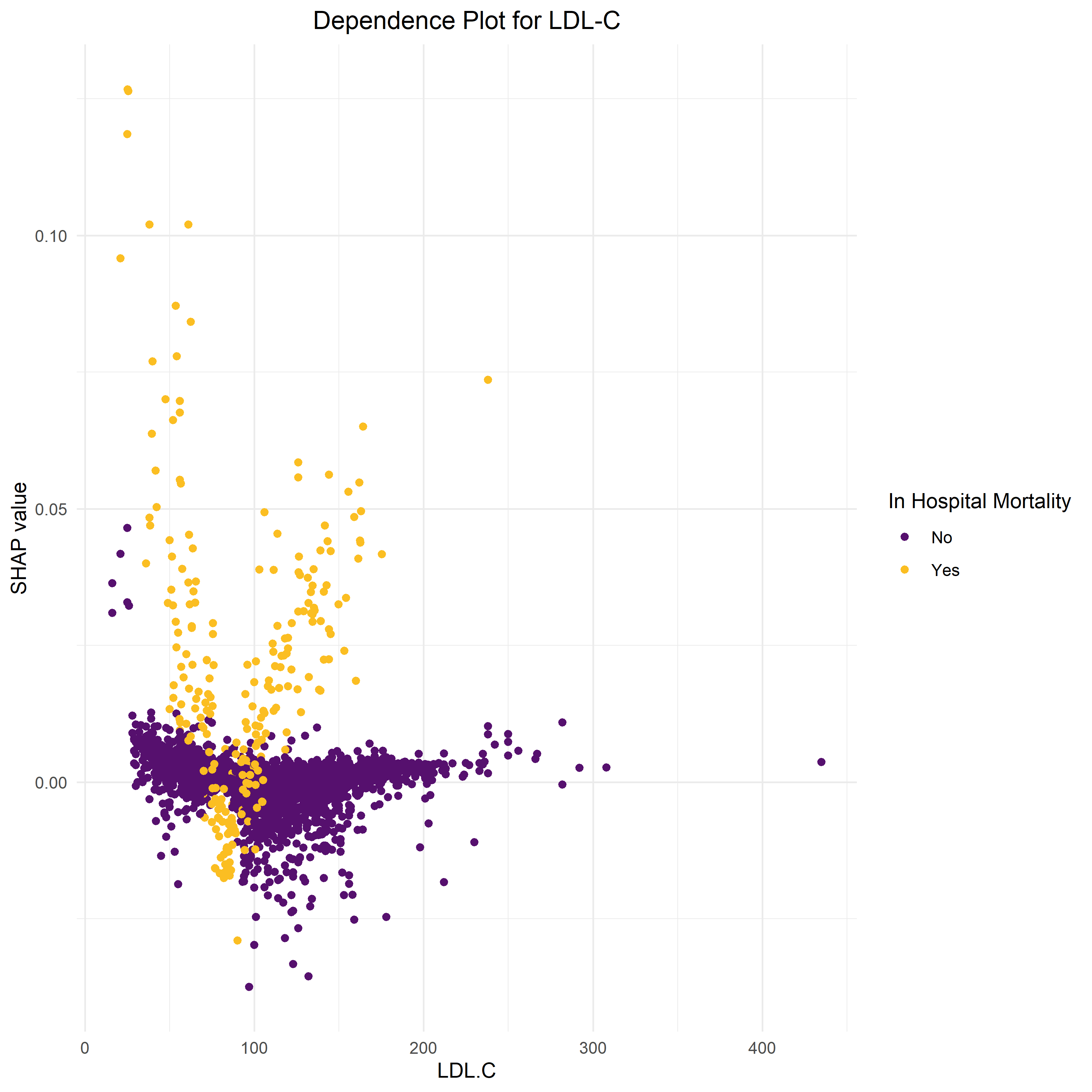


This plot shows the U-shaped relationship between LDL-C levels and SHAP values in predicting in-hospital mortality for AMI patients. Both lower and higher LDL-C levels are associated with increased SHAP values, indicating higher mortality risk at the extremes. Yellow dots represent patients who died in the hospital, while purple dots represent survivors.

##

## **Supplemental Figure 8.6.** SHAP dependence plot for LVEF.


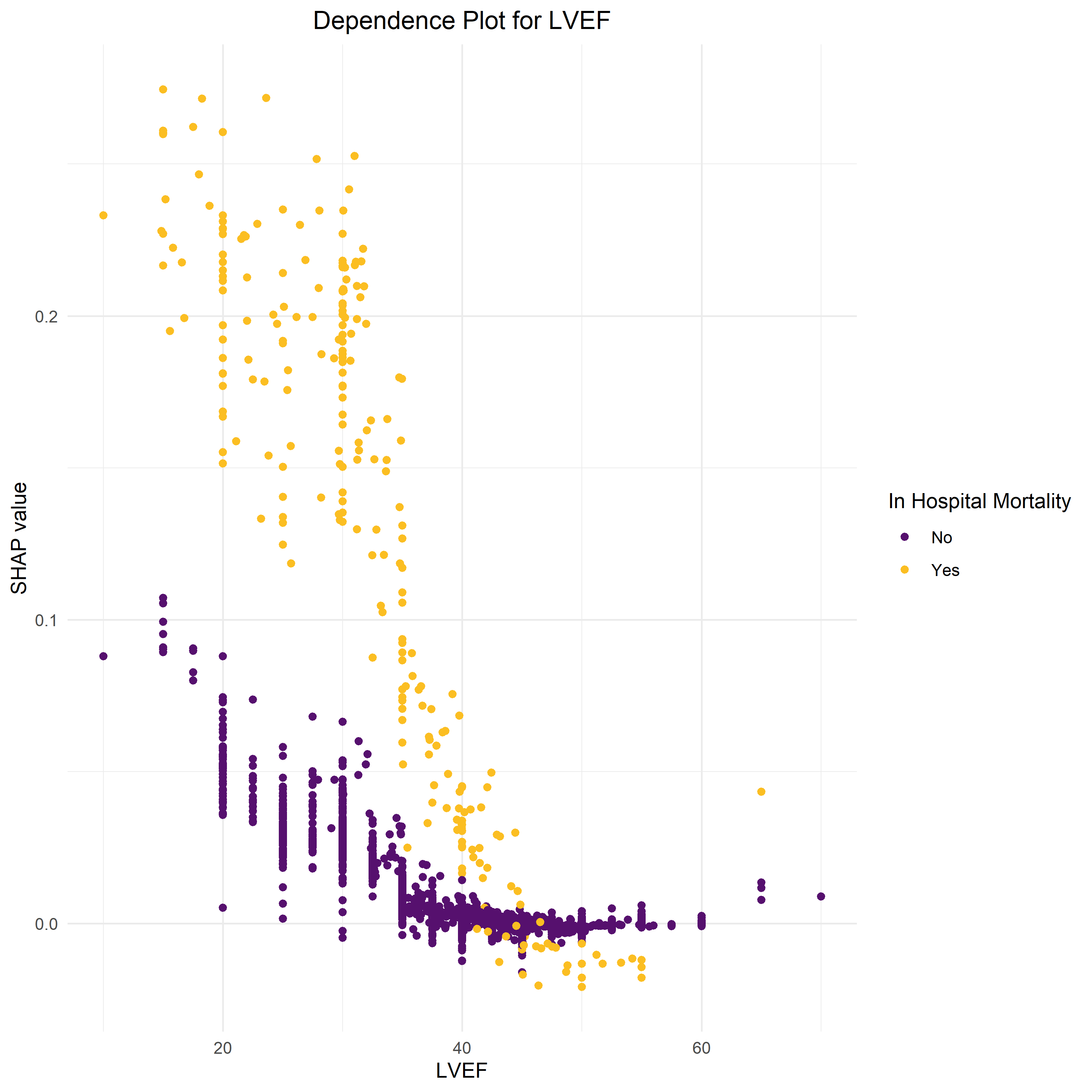


This plot shows the relationship between LVEF and SHAP values in predicting in-hospital mortality for AMI patients. Lower LVEF values are associated with higher SHAP values, indicating an increased mortality risk. Yellow dots represent patients who died in the hospital, while purple dots represent survivors.

## **Supplemental Figure 8.7.** SHAP dependence plot for total cholesterol.

This plot shows the U-shaped relationship between total cholesterol levels and SHAP values in predicting in-hospital mortality for AMI patients. Both lower and higher total cholesterol levels are associated with increased SHAP values, indicating higher mortality risk at the extremes. Yellow dots represent patients who died in the hospital, while purple dots represent survivors.


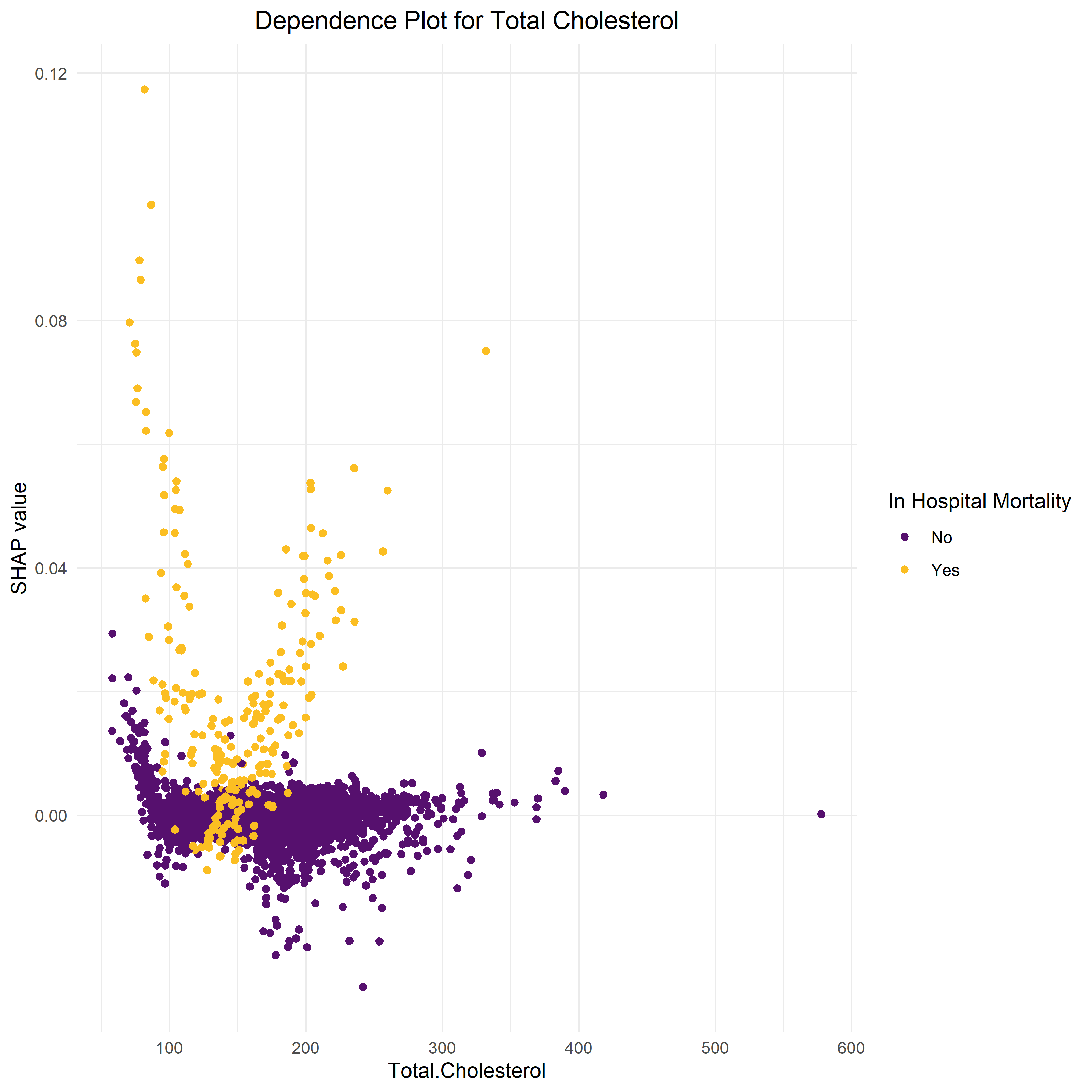


## **Supplemental Figure 8.8.** SHAP dependence plot for waist circumference.

This plot shows the relationship between waist circumference and SHAP values in predicting in-hospital mortality for AMI patients. Yellow dots represent patients who died in the hospital, while purple dots represent survivors.


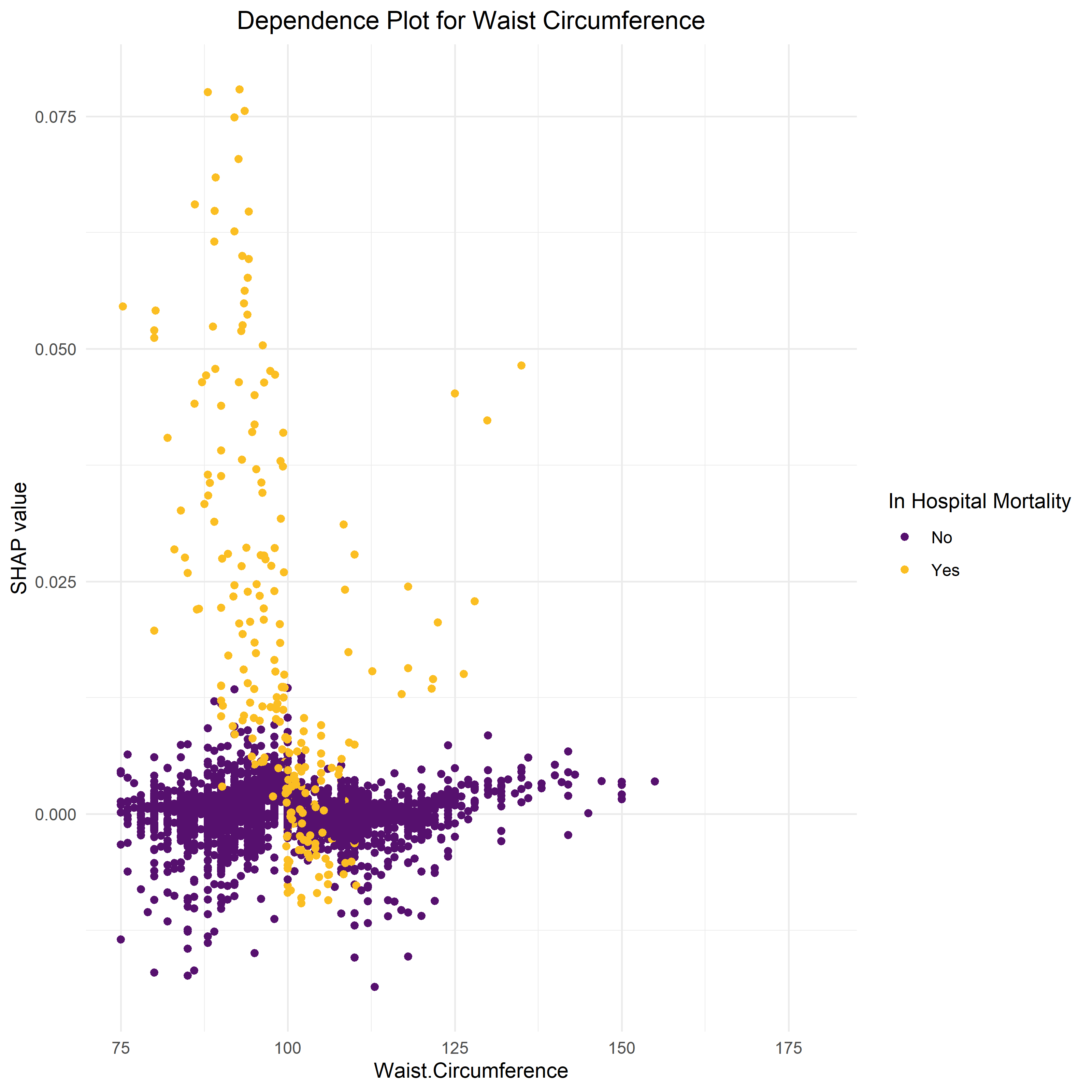


Sensitivity Analysis ___________________________________________________________________________

##
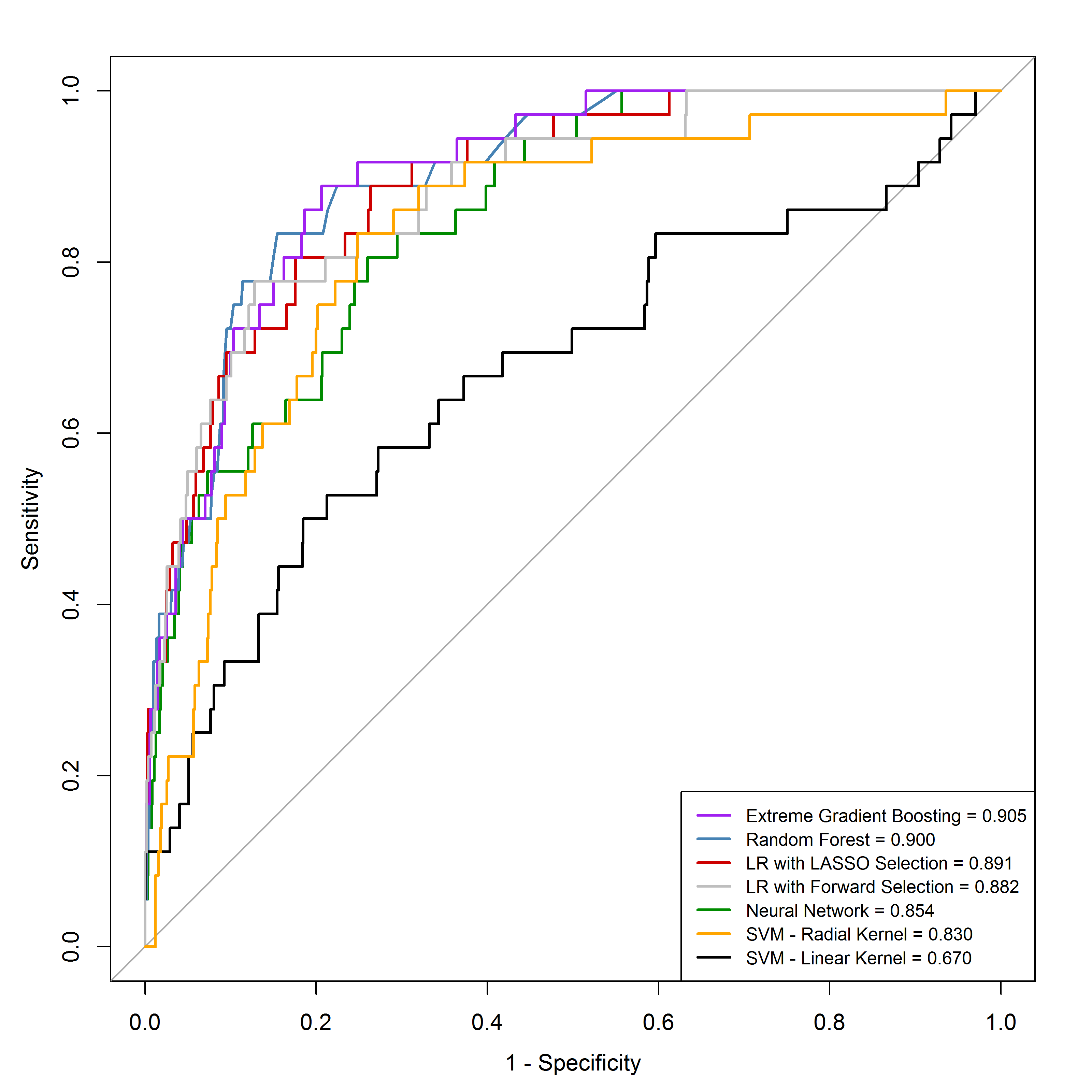
Supplemental Figure 9. Displaying the ROC Curves of Various Machine Learning Models in a subset of patients with STEMI (Sensitivity analysis).

This figure displays the AUC-ROC curves for machine learning models predicting in-hospital mortality in a subset of STEMI patients. Extreme gradient boosting achieved the highest AUC (0.905), followed closely by random forest (AUC = 0.900) and logistic regression with Lasso selection (AUC = 0.891). Logistic regression with forward selection recorded an AUC of 0.882, while the neural network achieved an AUC of 0.854. SVM with a radial kernel had an AUC of 0.830, and SVM with a linear kernel demonstrated the lowest performance with an AUC of 0.670.

## Supplemental Figure 10. Feature Importance of Variables Based on the Random Forest Model in a subset of patients with STEMI (Sensitivity analysis).


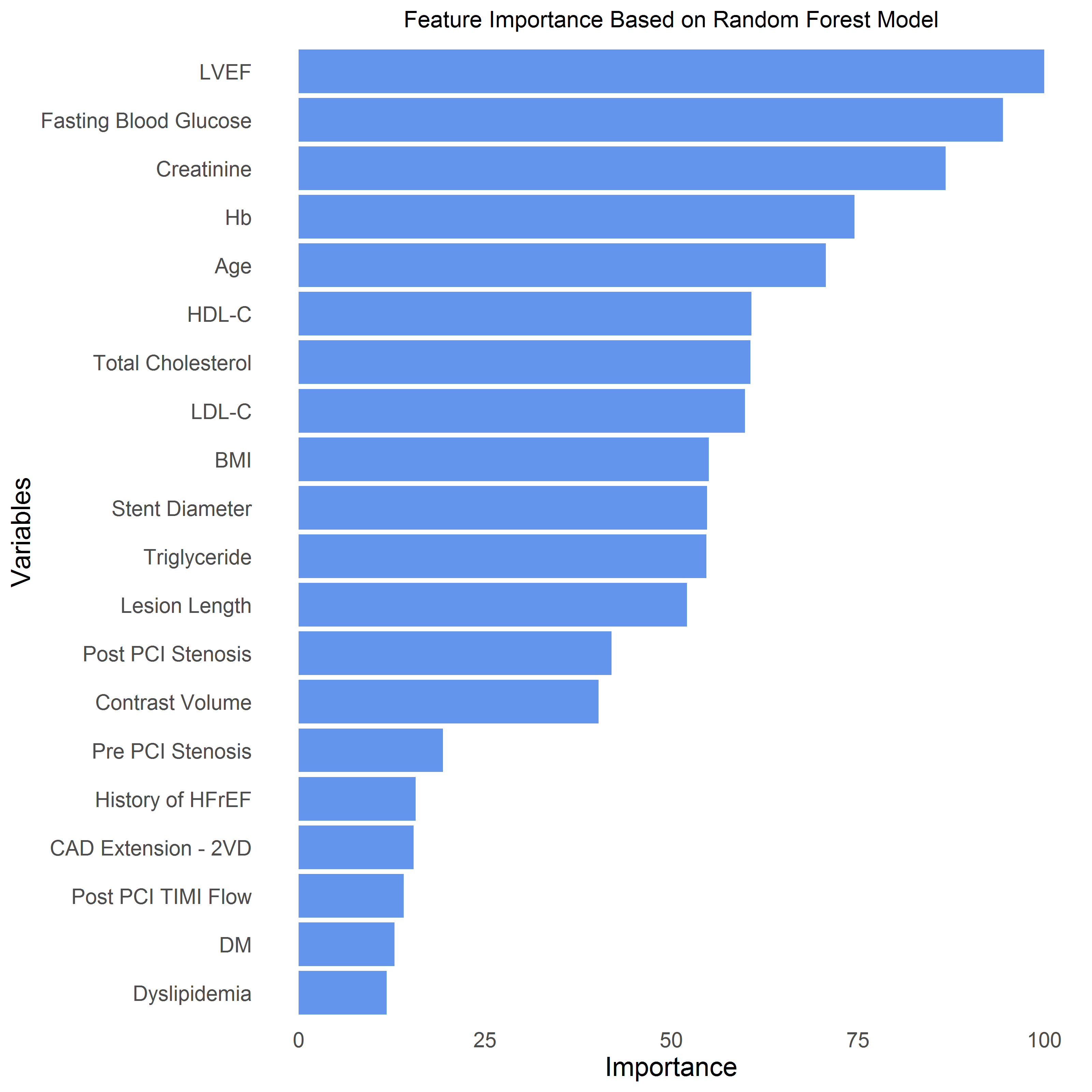


This bar chart illustrates the ranked importance of variables in the random forest model for predicting in-hospital mortality in a subset of STEMI patients. LVEF, fasting blood glucose, and creatinine were identified as the most influential predictors, followed by hemoglobin, age, and HDL-C. Additional important features include total cholesterol, LDL-C, BMI, stent diameter, and lesion length, among others.

##
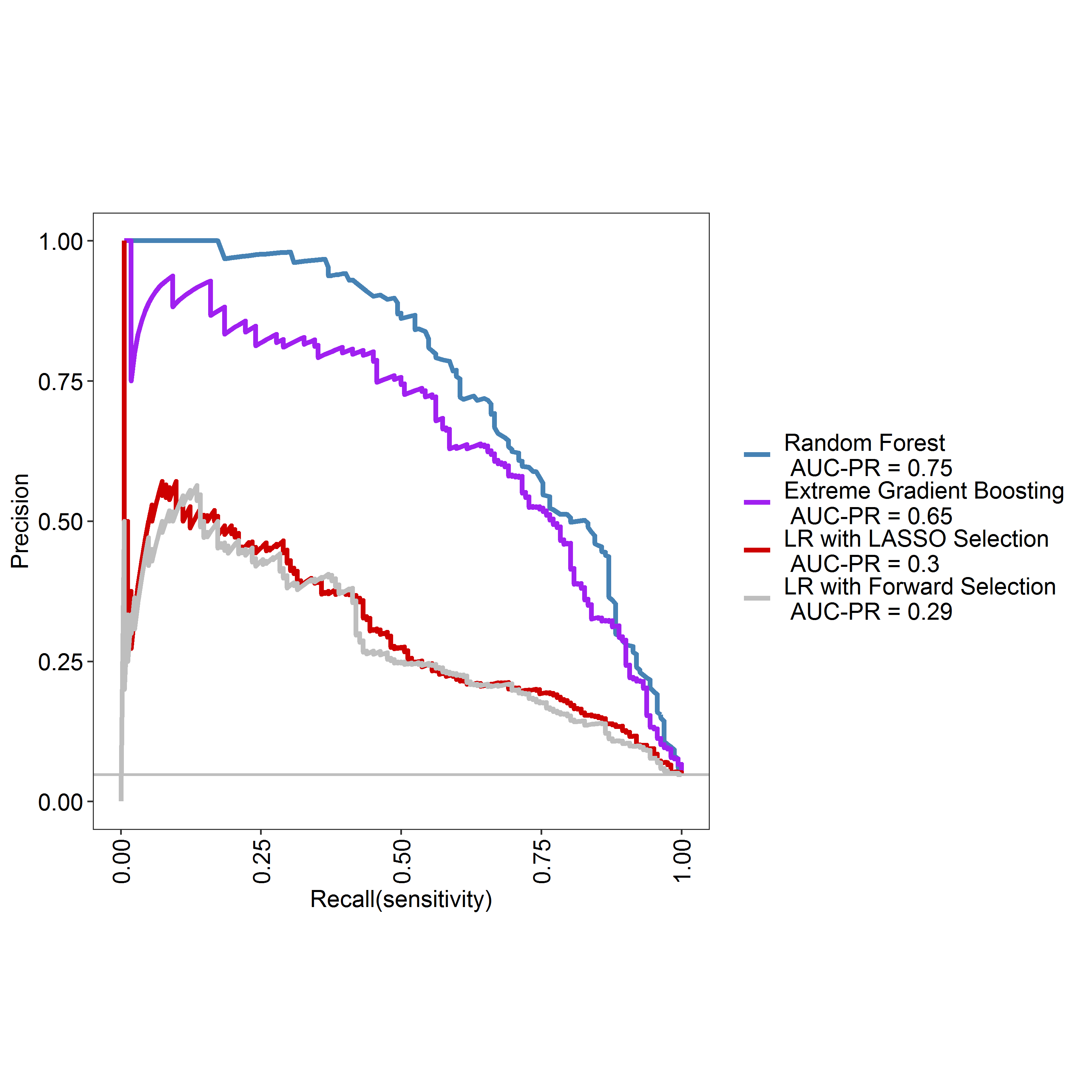
Supplemental Figure 11. Precision-recall curves of various machine learning models in a subset of patients with STEMI (Sensitivity analysis) (AUC-PR values shown in the figure).

##

This figure displays the precision-recall (PR) curves for various machine learning models predicting in-hospital mortality in a subset of STEMI patients. The random forest model achieved the highest AUC-PR (0.75), followed by extreme gradient boosting (AUC-PR = 0.65). Logistic regression with Lasso selection (AUC-PR = 0.30) and logistic regression with forward selection (AUC-PR = 0.29) demonstrated the lowest precision-recall performance.

##
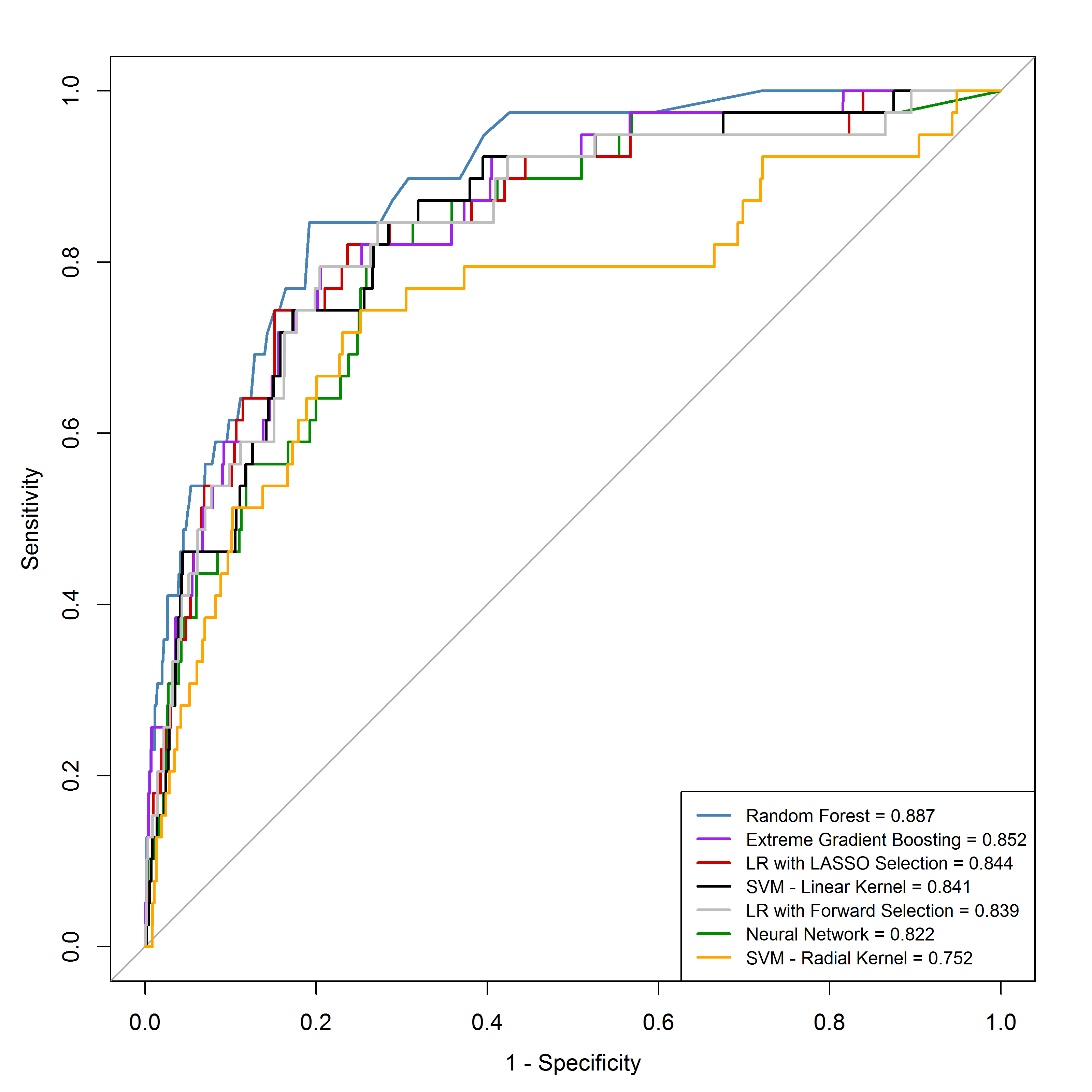
Supplemental Figure 12. Displaying the ROC curves comparing the performance of various models without applying ADASYN (AUC values shown in the figure).

This figure displays the AUC-ROC curves for various machine learning models predicting in-hospital mortality in AMI patients with the application of ADASYN for addressing class imbalance. The random forest model achieved the highest AUC (0.887), followed by extreme gradient boosting (AUC = 0.852) and logistic regression with Lasso selection (AUC = 0.844). Logistic regression with forward selection showed an AUC of 0.839, while the neural network model achieved an AUC of 0.822. SVM with a linear kernel and SVM with a radial kernel had the lowest performances, with AUCs of 0.841 and 0.752, respectively.

## Supplemental Figure 13. Cross-validation results for random forest performance across varying mtry values.


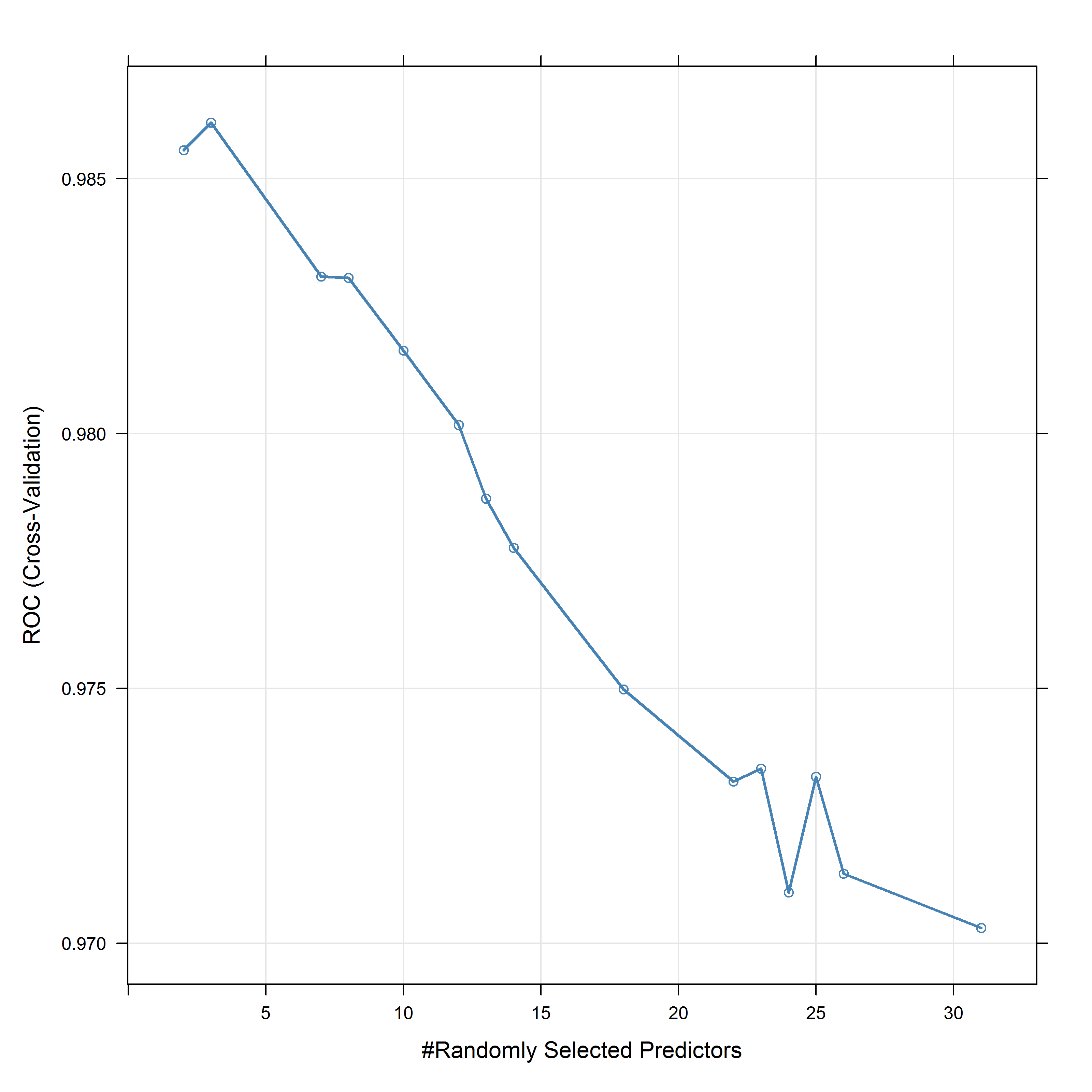


This plot illustrates the relationship between the number of randomly selected predictors at each split (mtry) and the cross-validated ROC performance for the random forest model. As mtrymtrymtry increases, a gradual decline in performance is observed, indicating that a smaller subset of predictors at each split yields better predictive accuracy.

## Supplemental Figure 14. Cross-validation results for random forest performance across varying ntree values.


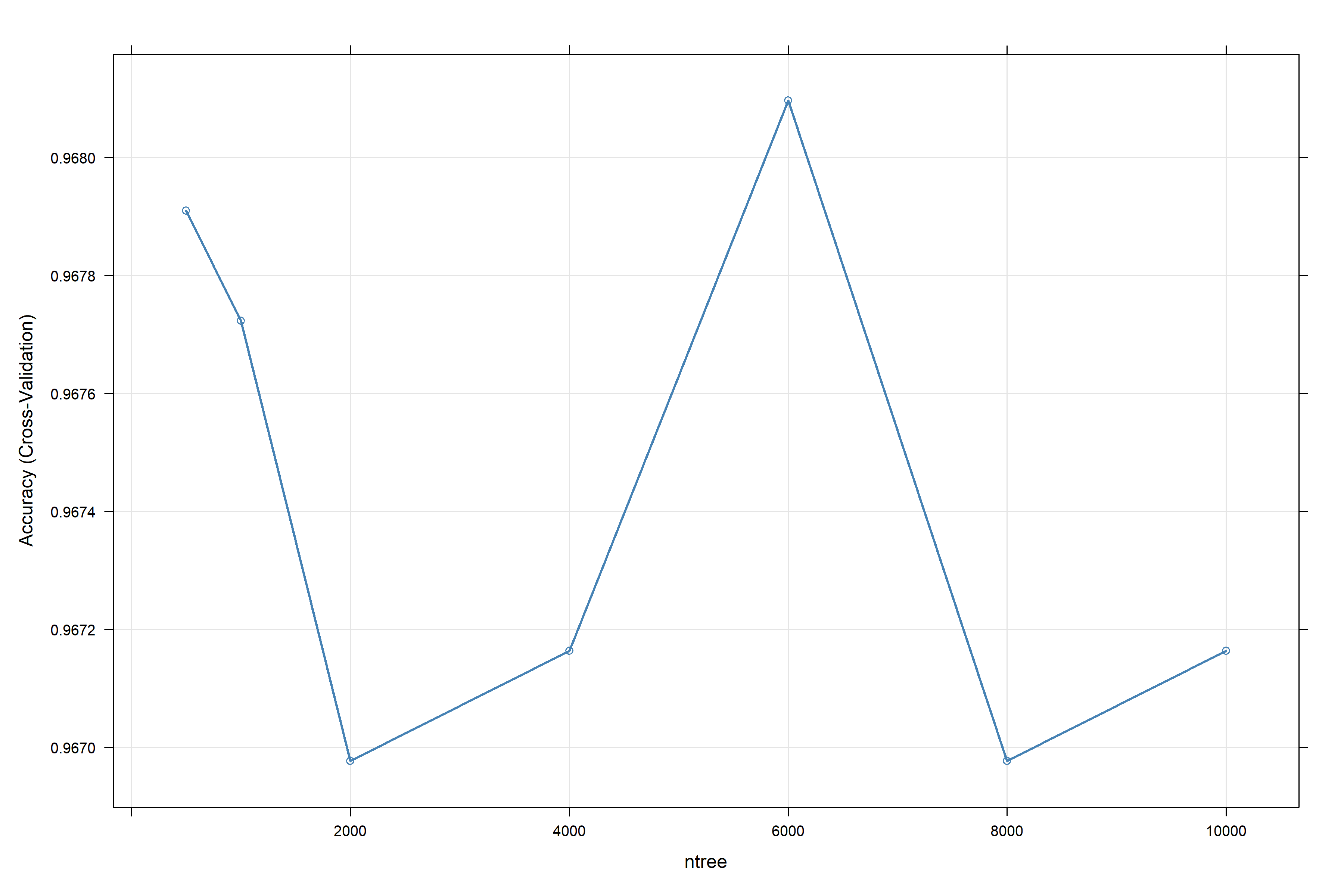


This plot demonstrates the accuracy of the random forest model across different numbers of trees (ntree) using cross-validation. The results reveal optimal performance near 6,000 trees.
